# Obstetric Facility Quality and Newborn Mortality in Malawi: A Cross-Sectional Study

**DOI:** 10.1371/journal.pmed.1002151

**Published:** 2016-10-18

**Authors:** Hannah H. Leslie, Günther Fink, Humphreys Nsona, Margaret E. Kruk

**Affiliations:** 1 Department of Global Health and Population, Harvard T.H. Chan School of Public Health, Boston, Massachusetts, United States of America; 2 Malawi Ministry of Health (IMCI), Lilongwe, Malawi; University of Manchester, UNITED KINGDOM

## Abstract

**Background:**

Ending preventable newborn deaths is a global health priority, but efforts to improve coverage of maternal and newborn care have not yielded expected gains in infant survival in many settings. One possible explanation is poor quality of clinical care. We assess facility quality and estimate the association of facility quality with neonatal mortality in Malawi.

**Methods and Findings:**

Data on facility infrastructure as well as processes of routine and basic emergency obstetric care for all facilities in the country were obtained from 2013 Malawi Service Provision Assessment. Birth location and mortality for children born in the preceding two years were obtained from the 2013–2014 Millennium Development Goals Endline Survey. Facilities were classified as higher quality if they ranked in the top 25% of delivery facilities based on an index of 25 predefined quality indicators. To address risk selection (sicker mothers choosing or being referred to higher-quality facilities), we employed instrumental variable (IV) analysis to estimate the association of facility quality of care with neonatal mortality. We used the difference between distance to the nearest facility and distance to a higher-quality delivery facility as the instrument.

Four hundred sixty-seven of the 540 delivery facilities in Malawi, including 134 rated as higher quality, were linked to births in the population survey. The difference between higher- and lower-quality facilities was most pronounced in indicators of basic emergency obstetric care procedures. Higher-quality facilities were located a median distance of 3.3 km further from women than the nearest delivery facility and were more likely to be in urban areas.

Among the 6,686 neonates analyzed, the overall neonatal mortality rate was 17 per 1,000 live births. Delivery in a higher-quality facility (top 25%) was associated with a 2.3 percentage point lower newborn mortality (95% confidence interval [CI] -0.046, 0.000, *p*-value 0.047). These results imply a newborn mortality rate of 28 per 1,000 births at low-quality facilities and of 5 per 1,000 births at the top 25% of facilities, accounting for maternal and newborn characteristics. This estimate applies to newborns whose mothers would switch from a lower-quality to a higher-quality facility if one were more accessible. Although we did not find an indication of unmeasured associations between the instrument and outcome, this remains a potential limitation of IV analysis.

**Conclusions:**

Poor quality of delivery facilities is associated with higher risk of newborn mortality in Malawi. A shift in focus from increasing utilization of delivery facilities to improving their quality is needed if global targets for further reductions in newborn mortality are to be achieved.

## Abstract 2

In a cross-sectional analysis, Hannah Leslie and colleagues examine the association between obstetric facility quality and newborn mortality in Malawi.

## Author Summary

**Why Was This Study Done?:**

- Large increases in access to health facilities in many low- and middle-income countries (LMIC) have not produced equivalent gains in newborn survival.
- Little data or evidence on the quality of delivery care in LMICs is currently available, and even less is known on the mortality impact of facility quality.

**What Did the Researchers Do and Find?:**

- We designed and implemented a study of neonatal mortality in Malawi, where facility delivery is highly prevalent and a quality assessment of all health facilities was conducted in 2013–2014.
- We quantified quality of maternal care at all delivery facilities based on 25 quality characteristics and classified the top 25% of facilities as higher quality. Average quality was low, with particular gaps in infrastructure and performance of basic emergency obstetric care procedures.
- We linked a nationally representative sample of 6,686 births between November 2011 and March 2014 to their delivery facility and used multivariable linear regression models to estimate the impact of quality on neonatal mortality. To overcome selection issues, we used the relative proximity of higher-quality facilities as an instrument for facility quality.
- Our empirical results suggest that delivering at a higher-quality facility is associated with a reduction of 23 deaths per 1,000 live births.

**What Do These Findings Mean?:**

- Expanded availability of health facilities does not guarantee access to essential elements of quality maternal and neonatal care.
- Improvements in facility quality could reduce newborn deaths substantially among women who would receive higher-quality care if it were more accessible.

## Introduction

Eliminating preventable infant mortality is a global health priority, reaffirmed in Sustainable Development Goal 3.2, which aims to reduce neonatal mortality to 12 per 1,000 live births by 2030 [1]. This is an ambitious goal: currently, over 2.5 million infants die each year in the first month of life [2]; neonatal mortality rates are estimated at 29 deaths per 1,000 live births in sub-Saharan Africa [3]. Globally, reductions in deaths within 28 days of birth have lagged decreases in postneonatal mortality. As a result, neonatal mortality now accounts for the largest share (44%) of under-5 mortality [2,4]. Achieving global targets in infant and child survival requires a redoubled focus on deaths in the first month of life.

Malawi was one of the few low-income countries to achieve the Millennium Development Goal (MDG) for child survival [5], a testament to high-level policy commitment to child health, donor-support for strengthening of health workforce capacity, and expanded maternal and newborn care [6]. Facility delivery rates increased from 53% in 2000 to 90% in 2014 [5], heavily influenced by a 2007 ban on deliveries with traditional birth attendants [7]. Although child mortality declined by more than 5% annually from 2000, newborn mortality declined less rapidly (3.3% per year) and remains 23 deaths per 1,000 live births. In response, the government of Malawi has recently adopted an Every Newborn Action Plan to end preventable newborn deaths [8].

Neonatal survival depends in large part on rapid and competent care during labor and delivery [6]. Basic neonatal resuscitation could avert as many as 30% of intrapartum-related newborn deaths [9]. An estimated 40% of deaths due to sepsis and tetanus could be prevented with infection control and hygienic cord care [10], and kangaroo mother care for low birth weight (LBW) infants should reduce neonatal mortality in these high-risk babies by half [6,11]. All of these interventions require qualified health workers as well as facility infrastructure and resources [6,12]. Simply delivering in a health facility does not guarantee care of sufficient quality to prevent newborn deaths [13–15]. A recent meta-analysis of 192 Demographic and Health Surveys (DHS) found inconsistent links between institutional delivery coverage and neonatal mortality [16]. Similarly, case studies in Rwanda and Malawi found no evidence of decreased neonatal mortality following large increases in facility-based delivery [7,17].

Research on the relationship between facility quality and mortality outcomes has been challenging not only because of generally scarce quality data in high-mortality settings but also because of the highly nonrandom selection of mothers with health complications into better-equipped referral facilities [16].

The aim of this study is to measure the association of quality of delivery care with neonatal mortality in Malawi. Malawi provides an ideal setting to test this association both because nearly all women deliver at a facility and because all health facilities in the country were recently assessed by a health facility census. The census allows us to identify all potential delivery locations for mothers and to construct relative distance measures. These measures enable us to employ instrumental variable (IV) estimation to better approximate the causal effect of facility quality on neonatal mortality. Determining whether facility quality is a barrier to reducing neonatal mortality in Malawi can inform policy there and in similar settings of persistently high neonatal mortality.

## Methods

### Ethical Approval

The original survey implementers obtained ethical approvals for data collection; the Harvard University Human Research Protection Program deemed this analysis exempt from human subjects review.

### Study Sample

Data on health facilities were obtained from the 2013 Service Provision Assessment (SPA), a census of health facilities conducted by the DHS program. The SPA includes an audit of facility resources, surveys on clinical practices, and direct observation of delivery in larger facilities.

Data on child survival were obtained from the 2013–2014 MDG Endline Survey (MES), a multiple indicator cluster survey (MICS) conducted in collaboration between the Malawi government and the United Nations Children’s Fund (UNICEF). The MES is a nationally representative household survey that employed a multi-stage stratified sampling strategy to identify households within enumeration areas (EAs) drawn from the 2008 census.

Spatial locations of all EAs in the MES were obtained from the Malawi National Statistical Office. We grouped facilities based on type and management authority in the SPA survey to create categories matching response options to the MES question on delivery location. We linked all women delivering in institutions to the nearest facility of the type in which she delivered (e.g., government hospital) by direct distance from the geographic centroid of her EA. Based on prior studies suggesting women are unlikely to deliver far from home [18–20], we excluded women matching to facilities over 50 km away, as these women were likely in another area for childbirth.

### Neonatal Mortality

Neonatal mortality was defined as death within the first 28 days of life [2] among all children born in the two years prior to interview date.

### Quality of Facility Delivery Care

We reviewed the framework of quality of care for pregnant women and newborns endorsed by the World Health Organization (WHO) [21] and identified domains characterizing provision of care at the ultimate delivery facility: infrastructure, human resources, essential supplies, and evidence-based practices in routine and emergency care. We then used the WHO Safe Childbirth Checklist in combination with existing evidence on interventions most likely to avert maternal and neonatal death [11,15,22,23] to identify 25 quality criteria available in the SPA survey (listed in Fig 2). In keeping with prior research [24], the overall quality score was based on the proportion of criteria met, with missing items excluded from the calculation of the score for that facility. Facilities were missing data for only two items: staff training (15% missing) and partograph use (1.9%). We classified a facility as a “higher-quality facility” if it met more than 18 of 25 criteria, corresponding to the 75th percentile of the quality score distribution for all delivery facilities.

We created an alternative quality metric for sensitivity analyses. For the subset of facilities with clinical observations, we combined the 25-item quality index with a validated metric of quality of process of intrapartum and immediate postpartum care from direct observation of deliveries (45 items total) [25].

### Covariates

We obtained data on socioeconomic status (household wealth index, educational attainment above secondary), maternal demographics (age, marital status), and pregnancy characteristics (parity, maternal age under 18, receipt of any antenatal care [ANC], and receipt of the minimum recommended four ANC visits) for each mother from the MES [26]. We also included other variables that have been shown to be associated with increased mortality risk: male gender, multiple birth, and LBW (defined as ≤2.5 kilograms or very small by maternal report if weight not available).

### Analysis Plan

We identified the SPA survey and MES sample in Malawi as a unique combination of data that permitted us to directly link facility quality to a population-representative sample of births. To address likely biases resulting from the nonrandom and unmeasured selection of more complicated deliveries into referral facilities, we selected IV analysis as the appropriate empirical strategy. We chose relative distance to quality care as the instrument based on existing health systems research in high-income settings [27–30]. Key domains of maternal care quality were identified from global guidelines following prior analytic work [24]; we refined this index after receiving the data based on the specific indicators available in the Malawi SPA survey. We prespecified an additive summary measure, as is standard practice in this field [31], and focused on a binary quality indicator for simplicity in our main empirical model. Given that clear and objective thresholds for sufficient quality are not currently available, we classified the top 25% of all facilities in our sample as higher-quality in our initial model and then explored two alternative cutoffs as well as the continuous quality score. We conducted an exploratory assessment of the shape of the relationship between quality and mortality, defining higher quality as the top 75%, top 50%, and top 10% of facilities in turn.

### Statistical Analysis

We present separate descriptive statistics for delivery facilities and births. Delivery facilities were defined as SPA facilities offering delivery services with at least one birth in the MES sample. Maternal and infant characteristics were weighted by the MES women’s sampling weight, rescaled to the analytic sample. We describe mortality by region and facility type and assess significance using an *F*-test corrected for clustering.

We first modeled mortality against delivering in a higher-quality facility in unadjusted linear regression. As we anticipated unmeasured selection of complicated deliveries into referral facilities would bias the relationship between delivery in a higher-quality facility and newborn survival, we employed IV analysis using the difference between distance to the nearest delivery facility and distance to a higher-quality facility as the instrument. We selected this instrument on the assumption that, for a given level of remoteness from the health system, the relative location of a higher-quality facility is random. By using differential distance rather than direct distance to quality care, we explicitly account for systematically higher health risks related to living in areas with limited access to the health system.

To be a valid instrument, differential distance must relate to mortality only through facility quality and not through a direct causal link or any shared common causes. Based on the distribution of measured confounders across contextual factors, we identified urban location and health system density as key control variables to eliminate other possible links between differential distance and neonatal outcomes. Health system density was defined as the natural log of one plus the number of health facilities within 20 km of the center of the EA.

The IV analysis estimates a local average treatment effect (LATE), i.e., the effect of delivering in a higher-quality facility among women whose choice is affected by relative distance [32]. We present further details on the causal model, an assessment of the underlying assumptions, falsification tests [33], and estimation of bounds for the LATE estimate if assumptions do not hold in the Supporting Information (S1–S3 Texts).

We plotted predicted probability of delivering in a higher-quality facility and of neonatal mortality against differential distance using a fractional polynomial plot to visualize the relationships among distance, quality, and mortality. We used two-stage least squares to fit a linear probability model of mortality on delivering in a higher-quality facility; linear probability models are standard in IV analysis [29]. In addition to urban residence and density of the health system, we controlled for maternal socioeconomic status and maternal and infant characteristics associated with mortality to increase precision in the estimate [33]. Observations with missing covariates (18, 0.3%) were excluded from the analysis. All analyses accounted for stratified sampling and clustering within EAs.

We performed several robustness checks on the measurement of quality. To assess sensitivity to the threshold chosen for high quality, we (A) increased the threshold to an absolute standard of 0.80 of 1.00 score on the quality index, (B) lowered the threshold of high quality to include the top tertile of facilities, and (C) employed the continuous standardized quality index in place of the binary indicator of high quality. To check the measure construction, we applied principal components analysis (PCA) to create a weighted summary of the 25 items. To validate the content used to construct the quality metric, we employed the composite index described above that included direct observation of deliveries, the gold standard of clinical quality measurement. This analysis was limited to the facilities where observations occurred.

We conducted two additional analyses to assess whether simpler measures of quality would show the same relationship as the facility quality index. We used hospital delivery as the exposure and differential distance to nearest hospital as an instrument. Secondly, we measured overall facility capacity using seven indicators of scope of services available [7] and used this index to define higher-quality facilities (top 25%) and to calculate differential distance to such facilities. We repeated all analyses using a probit model, which bounds the outcome between 0 and 1, to compare with the findings of the linear probability model.

## Results

**Fig 1:**
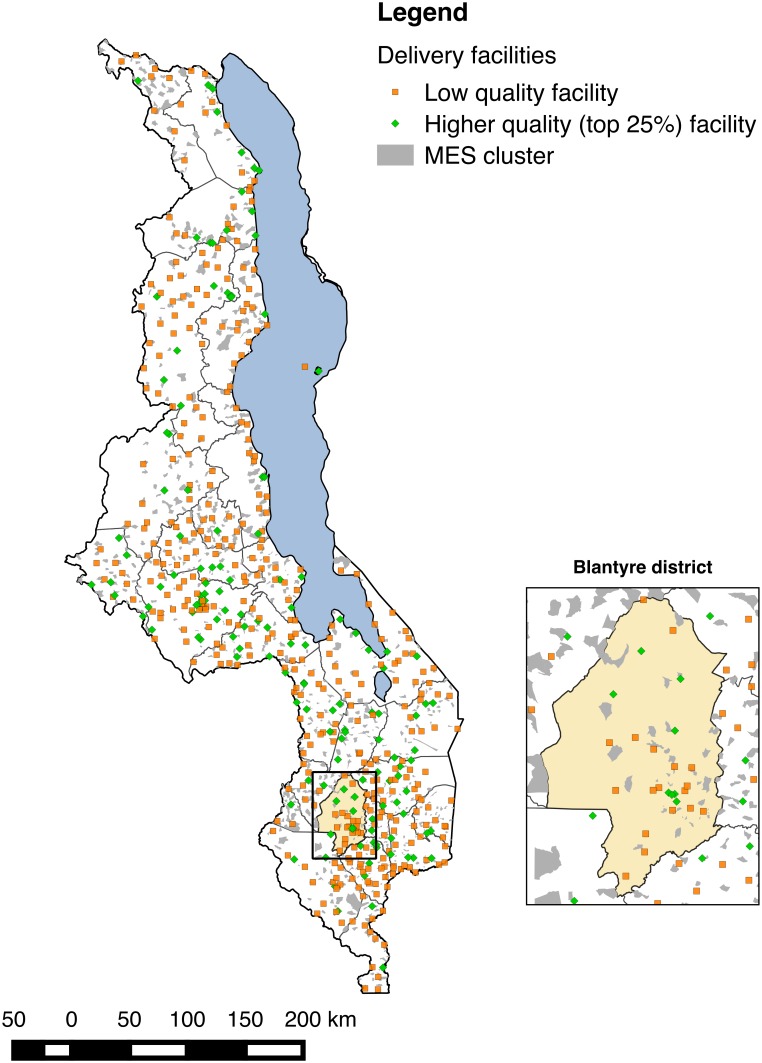
Distribution of health facilities in Malawi relative to MES enumeration areas and magnification of Blantyre district and city.

The SPA assessed 977 of 1,066 health facilities in Malawi (92.2% response rate); 3% of facilities refused assessment, while the remainder were closed, empty, or inaccessible. Delivery services were provided by 540 facilities in total. The MES interviewed 24,230 of 25,430 eligible women (95.3% response rate), 7,576 of whom reported giving birth in the two years preceding the survey. Fig 1 shows the distribution of MES clusters and health facilities throughout Malawi; EAs are by construction small, with a target population of approximately 1,000 and an average size of 6.7 km^2^. Most women (6,935, 91.5%) reported a facility-based delivery; of these, 160 reported a facility that could not be matched to the SPA facility types, 102 lived in EAs that we were not able to match to census EAs, and 138 were matched to delivery facilities over 50 km away. The analytic sample comprised 6,535 women with live births (6,686 neonates with twins) matched to 467 unique delivery facilities; 6,668 neonates with complete data on covariates were retained in regression analyses.

Table 1 provides characteristics of delivery facilities. The majority of delivery facilities were health centers or clinics, with medical assistants and clinical technicians most likely to be the highest qualified clinician. One hundred thirty-four facilities met the threshold of higher quality (top 25% of the total 540 delivery facilities, equivalent to at least 18 of 25 items fulfilled). This included the majority of hospitals but only 16% of health centers; higher-quality facilities had larger (average of 73 clinical personnel versus 19) and more highly trained staff. The average quality score at the top 25% of facilities was 0.80 compared to 0.56 at all other facilities.

**Table 1:** Characteristics of delivery facilities in study sample (*n* = 467).

	All facilities (*n* = 467)		Lower-quality facilities (*n* = 333)		Higher-quality facilities (top 25%) (*n* = 134)	
	*n* or mean	% or SD	*n* or mean	% or SD	*n* or mean	% or SD
Urban	73	15.6%	28	8.4%	45	33.6%
Public	138	29.6%	81	24.3%	57	42.5%
Facility type						
Central hospital	4	0.9%	0	0.0%	4	2.8%
District hospital	24	5.1%	0	0.0%	24	17.0%
Other hospital	64	13.7%	17	5.2%	47	33.3%
Health center/clinic	375	80.3%	316	96.9%	59	41.8%
Highest clinician on site						
Medical doctor	75	16.1%	7	2.1%	68	48.2%
Assistant medical officer	8	1.7%	5	1.5%	3	2.1%
Clinical officer	350	74.9%	292	89.6%	58	41.1%
Registered nurse	4	0.9%	3	0.9%	1	0.7%
Enrolled nurse	29	6.2%	25	7.7%	4	2.8%
Other	1	0.2%	1	0.3%	0	0.0%
Clinical staff (mean, SD)	34.79	50.84	19.28	12.17	73.34	81.16
Maternity beds (mean, SD)	11.91	13.20	8.11	4.27	21.58	21.05
Quality domains^1^						
Infrastructure and staff (mean, SD)	0.50	0.23	0.41	0.18	0.73	0.16
Delivery supplies (mean, SD)	0.65	0.18	0.59	0.16	0.78	0.16
Routine care practices (mean, SD)	0.71	0.14	0.68	0.14	0.80	0.10
Basic emergency care procedures (mean, SD)	0.56	0.25	0.46	0.21	0.82	0.16
Quality of maternal care (mean, SD)	0.63	0.14	0.56	0.10	0.80	0.07

^1^ Each quality domain is the average of the items detailed in Fig 2: seven items for infrastructure and staff, five for delivery supplies and medications, six routine clinical practices, and seven emergency clinical practices.

SD: standard deviation

**Fig 2:**
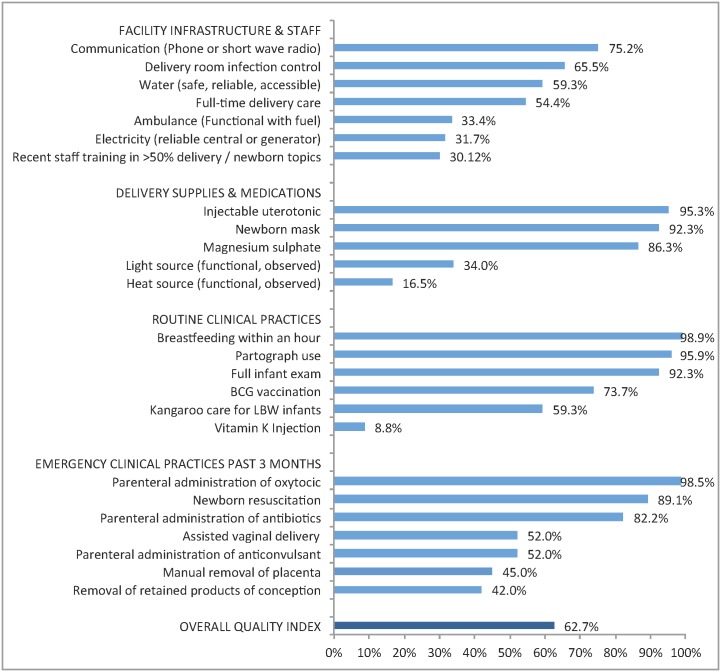
Performance on delivery facility quality index: percentage of facilities with key resources and services (*n* = 467). Legend: BCG, Bacille Calmette-Guérin vaccine; LBW, low birth weight. Sixty-two facilities (13.3%) were missing data on staff training and eight (1.7%) were missing data on partograph use. Percentages shown are out of facilities with non-missing data per indicator.

Fig 2 details the performance of delivery facilities on the facility quality index. The average facility achieved approximately 16 of the 25 items on the quality index (63%), with notable deficiencies in key infrastructure as well as selected supplies. Facilities commonly reported routine clinical practices (immediate breastfeeding, partograph use, and full infant exam all >90%), although vitamin K injections were rare. Nearly all facilities reported performing at least one basic emergency procedure in the past three months. As shown in Table 1, the difference between higher and lower quality was most pronounced in performance of basic emergency obstetric care, with a difference of over 40 percentage points.

Table 2 presents the women’s study sample: most women were rural dwellers, married, and with basic education (19.0% secondary education or more). Access to the health system was high: 99.5% of women attended at least one ANC visit, the average number of facilities within 20 km was 24.3 (median 13), and the average distance to matched delivery facility was 8.4 km (median 6.0 km). Women with greater educational attainment, primiparous women, and women carrying multiple infants or LBW infants were more likely to deliver in higher-quality facilities. A total of 115 neonatal deaths were reported, with higher mortality rates at higher-quality facilities. Mortality rates were similar between urban and rural areas (17.9 versus 18.3 deaths per 1,000) as well as at public and private facilities (18.7 versus 14.9 deaths per 1,000) but significantly higher in hospitals than non-hospitals (24.2 versus 14.3 deaths per 1,000).

**Table 2:** Descriptive statistics of women and infants in study sample.

	Total		Delivery at lower-quality facilities			Delivery at high-quality facilities		
	*n* or mean	% or SD	*n* or mean	% or SD		*n* or mean	% or SD	
**Women's characteristics**	Weighted *n* = 6,535		Weighted *n* = 3,641			Weighted *n* = 2,894		
**Demographics**								
Urban	839	12.8%	327		9.0%	512		17.7%
Household has improved water source	5,605	85.8%	3,118		85.6%	2,487		86.0%
Household has access to a toilet	506	7.7%	217		6.0%	289		10.0%
Age at delivery (mean, SD)	26.20	6.56	26.24		6.56	26.15		6.56
Secondary education or above (*n* = 6,534)	1,242	19.0%	580		15.9%	662		22.9%
Marital status (*n* = 6,533)								
Currently married	5,529	84.6%	3,127		85.9%	2,402		83.0%
Formerly married	745	11.4%	398		10.9%	347		12.0%
Never married	259	4.0%	113		3.1%	146		5.0%
**Risk factors**								
Age <18 at delivery	825	12.6%	439		12.1%	386		13.3%
Parity (mean, SD)	3.30	2.11	3.38		2.11	3.20		2.10
ANC visits—any	6,505	99.5%	3,629		99.7%	2,875		99.4%
ANC visits—at least four	3,074	47.6%	1,690		47.0%	1,383		48.5%
**Delivery characteristics**								
Delivery location								
Government hospital	1,826	27.9%	128		3.5%	1,697		58.6%
Government health center	3,880	59.4%	3,188		87.5%	692		23.9%
Private facility	150	2.3%	103		2.8%	46		1.6%
CHAM/Mission hospital	402	6.1%	20		0.6%	382		13.2%
CHAM/Mission health center	279	4.3%	201		5.5%	77		2.7%
Health facilities within 20 km (mean, SD)	24.33	28.89	21.34		27.58	28.09		30.03
Distance (km) to nearest facility of delivery type (mean, SD)	8.42	7.96	7.00		6.43	10.22		9.24
**Infant characteristics**								
	Weighted *n* = 6,690		Weighted *n* = 3,709			Weighted *n* = 2,981		
Male	3,414	51.0%	1,935	52.2%		1,479	49.6%	
First birth	1,597	23.9%	827	22.3%		770	25.8%	
Multiples	307	4.6%	135	3.6%		172	5.8%	
Low birth weight (*n* = 6,676)	1,062	15.9%	555	15.0%		507	17.0%	
Unintended (*n* = 6,688)	2,965	44.3%	1,702	45.9%		1,262	42.3%	
Outcome: Death within 28 days	115	1.7%	47	1.3%		68	2.3%	

Sample restricted to women delivering in an institution that plausibly matched an institution in SPA (<50 km to facility of delivery type) and weighted using women’s sampling weight scaled to effective sample size. Infant sample size is higher than women’s sample size due to twins.

CHAM: Christian Health Association of Malawi; SD: standard deviation

**Fig 3:**
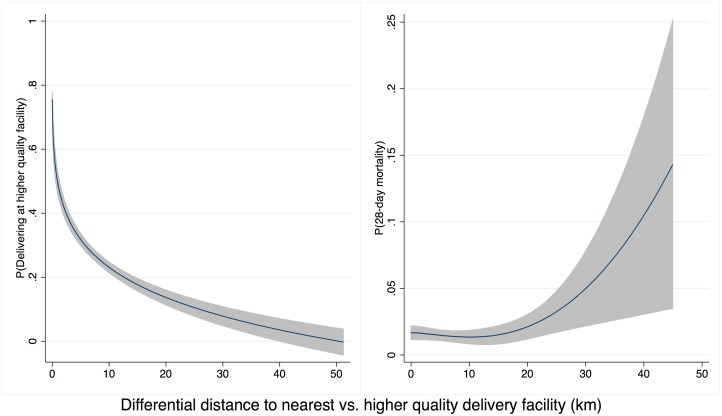
Distance to high-quality facility and (A) delivery in high-quality facility (B) neonatal mortality. Legend: Predicted values from a fitted fractional polynomial (degree 2) of distance against delivering at a high-quality facility (A) and neonatal mortality (B), with 95% CI, weighted using scaled sampling weight for each woman.

Higher-quality delivery care was less accessible than any delivery care: the closest higher-quality facilities were on average 6.2 km (median 3.3 km) farther from households than the nearest delivery facility of any quality. Differential distance to a higher-quality facility was strongly negatively associated with delivery at a higher-quality facility. As shown in Fig 3A, the probability of delivery at a high-quality facility declined from 75% for women where the closest facility was a higher-quality facility (1,623 of 2,152) to 7% for women with a higher-quality facility more than 30 km more distant than the closest low-quality facility (7 of 105). The probability of neonatal mortality increased as the additional distance to higher-quality care increased, although considerable uncertainty exists above 20 km (Fig 3B).

The unadjusted regression model suggested a 0.6% point linear increase (95% CI -0.1%, 1.3%) in the probability of neonatal death for delivery in higher-quality facilities (Table 3). This estimate of nonsignificantly increased risk for infants born at better facilities applies to the full population of facility births but does not account for maternal factors or selection into such facilities. Although not statistically significant, this positive association is consistent with risk selection, in which sicker women and neonates are referred to better facilities. In the fully adjusted IV analysis, the estimated impact of delivering at a high-quality facility on neonatal mortality was -0.023 (95% CI -0.046, 0.000, *p* = 0.047). The predicted prevalence of neonatal mortality was 28.3 deaths per 1,000 (95% CI 16.8, 39.8) in lower-quality facilities compared to 5.2 deaths per 1,000 (95% CI -0.7, 17.4) for delivery in higher-quality facilities, holding all covariates at their mean values. The IV estimate improves on the regression estimate by accounting for confounding between facility quality and mortality; it applies to the subset of women who would receive higher-quality care if it were more accessible.

**Table 3:** Regression results for the association between high-quality delivery facility and neonatal mortality (*n* = 6,668)^1^.

Model	F test of IV strength	β (95% CI)
**Main models**		
Unadjusted ordinary least squares	NA	0.006 (-0.001, 0.013)
Instrumental variable^2^	273.6	-0.023 (-0.046, <-0.001)
**Robustness assessment of instrumental variable model**		
Modifying the threshold of high quality		
1. Absolute threshold (≥0.80 of 1.00) for classifying facilities as high quality^2^	310.7	-0.030 (-0.058, -0.003)
2. Lower threshold (top 33%) for classifying facilities as high quality^2^	288.6	-0.033 (-0.058, -0.007)
3. No threshold: continuous quality index, standardized^2^	95.9	-0.027 (-0.054, <0.001)
Modifying the calculation of the quality index		
4. Weighted summary of quality items using PCA^2^	278.5	-0.026 (-0.048, -0.003)
Modifying the quality index to include data from clinical observations		
5. Alternative quality metric: facility quality and clinical quality of observed deliveries (20 additional items)^2^ (*n* = 4,171)	328.1	-0.016 (-0.038, 0.005)

^1^ Eighteen observations with missing values on covariates (17 for infant birth weight, 1 for maternal education) excluded from all analyses.

^2^ Adjusted for the following: urban, logged number of facilities within 20 km, wealth index quintiles, maternal secondary education, maternal age <18, male infant, multiple infant, LBW infant, primiparous mother.

IV: Instrumental variable; PCA: Principal components analysis

Tests of IV assumptions are reported in detail in the Supporting Information. Differential distance was strongly associated with quality of delivery facility (S1 Table). Infant and maternal risk factors were relatively evenly distributed across the range of differential distance (S2 Table), and falsification tests did not reject differential distance as a valid IV (S3 Table), lending support to the exclusion restriction and assumption of no unmeasured confounding of instrument and outcome. However, estimation of bounds around the IV estimate, should identifying assumptions not be met, showed a high degree of uncertainty, inclusive of the null (S4 Table).

Robustness results are shown in Table 3. In all specifications, including the main model, differential distance was strongly associated with delivering in a higher-quality facility, well above minimum thresholds for instrument strength [34,35]. Altering the threshold for higher quality using a continuous quality metric or calculating a weighted summary for the quality metric did not change the results (Models 1–4). Combining the facility quality index with a validated metric of quality of the process of care as directly observed resulted in a weaker association with mortality, -0.016 (95% CI -0.038, 0.005), although this analysis was limited to a smaller, higher-quality set of facilities.

Additional analyses employing simpler quality metrics resulted in estimates of association near -20 deaths per 1,000 with wide CIs inclusive of the null, suggesting such metrics are too coarse to fully capture meaningful variation in quality (S5 Table). In exploratory assessment of linearity of the relationship between quality and mortality, there were no significant protective associations of more lenient definitions of higher quality; the protective association obtained in the main model held true using a stricter categorization of higher quality (S6 Table). Results for the main model and all sensitivity analyses were unchanged in probit models (S4 Text).

## Discussion

This study is, to our knowledge, the first to link nationally representative data on births to detailed data of delivery facility quality in a sub-Saharan African setting. Our results suggest that delivery facilities in Malawi are both accessible and highly utilized, but that facility quality falls substantially short of global standards of evidence-based care. We found that higher-quality facilities, in the top 25% of our quality scale, were associated with 23 fewer neonatal deaths per 1,000 live births than other facilities in Malawi. This suggests improvements in facility quality could reduce mortality substantially among women who would deliver in higher-quality facilities were such facilities available.

Even though large improvements in neonatal survival seem plausible with high-quality care, the estimated reduction in mortality is large and may not necessarily be generalizable to other settings, including the full population of Malawi. The IV estimates shown represent LATEs, i.e., the causal effect (if all assumptions are met) of getting access to high-quality care in the subpopulation of women prevented from using such facilities by the relative distance. Large relative distances are more likely in rural and less developed areas, where baseline mortality is higher and potential improvements more substantial. The average population effect of quality will likely be smaller than the association estimated here, particularly as some women will always deliver in higher-quality facilities, whether by choice or referral.

Quality of care is increasingly recognized as central to the post-MDG global health agenda [36]. However, few prior studies have been able to move beyond access to care to systematically quantify quality of care [19]; most prior research on quality of care and maternal and neonatal outcomes consists of evaluations of specific quality improvement interventions [11]. This study extends existing knowledge by considering quality of delivery care at the facility level for the entire health system and by assessing the relationship of quality to mortality rather than intermediate health indicators.

A key strength of this study was the ability to link detailed data on health facility quality with population-representative mortality data. The detailed spatial location data from both surveys allowed us to construct relative distance instruments, which provided a means of estimating the causal relationship between quality and mortality despite salient selection concerns. Our main findings were robust in multiple sensitivity analyses. Finally, we found that although simpler quality indicators supported the generally protective association of quality, they did not capture the full variability of delivery care that may be important to newborn survival.

The study had several limitations. Women could have been matched incorrectly with facilities based on error in facility classification or location data. However, few women were matched to facilities implausibly far from their location, strengthening the credibility of the match. The small size of EAs mitigates the magnitude of misclassification due to displacement between a woman’s home and the EA center. Any misclassification that did occur would likely introduce greater error in estimation and bias results towards the null. A second potential limitation is the high variability in results of IV analyses; based on guidance in the literature, the sample size and strength of the instrument in this analysis should have been sufficient for the IV to be less biased than linear regression on average [37]. In addition, IV analysis depends upon assumptions, such as the exclusion restriction and lack of unmeasured confounding, that can be falsified but never fully verified. Extensive testing of the instrument provided support for the analysis while indicating that the resulting estimates depend critically on these assumptions. Fourth, our analysis did not address interpersonal quality of care, which could shape women’s choice of delivery facility [18,38]. Finally, an alternative to the facility quality index incorporating direct clinical observation showed a weaker association with mortality, as did analyses with coarser quality measures. Given the smaller sample sizes with the larger quality scale, it is hard to directly compare these estimates; the lack of significance in models with a larger number of items could reflect insufficient power or the diminished variation in quality among facilities with more extensive assessments.

Further research is needed to affirm and extend these findings. Validated, efficient metrics of facility quality are essential to strengthen and extend this area of inquiry. Identification of the minimum quality of care sufficient to ensure health outcomes is a particularly critical need in global health research. Replication in countries with higher mortality burdens and different health system capacities would strengthen the generalizability of these results. Such an undertaking is potentially feasible where detailed facility assessments have occurred prior to population health studies that include location data. Multiple tools for facility assessment have been employed throughout sub-Saharan Africa [39] in addition to the more commonly used population health surveys, yet their use for research has been limited to date. In general, linking facility surveys to population outcomes is complicated by the random sampling used for facility assessments and by displacement of household locations to preserve individual anonymity [40]. Full national facility censuses with quality assessments like the one conducted in Malawi would allow more research linking household heath behaviors and outcomes to facility indicators.

What do these findings imply for policy? Malawi is a leader in sub-Saharan Africa in implementing evidence-based policies to improve maternal and child health; the recently adopted Every Newborn Action Plan explicitly identifies improving facility quality as one means towards reducing newborn mortality [8]. This study provides strong and direct empirical support for such a policy and should galvanize targeted quality improvement interventions to extend child survival gains to newborns. Critical infrastructure and performance of basic emergency obstetric care functions may be priority areas for improvement. Neonatal mortality rates vary widely by district from under 15 to over 40 per 1,000 live births in urban versus rural districts [8]. In this context, the findings suggest targeted interventions at facilities in areas with no high-quality facilities, particularly in high-mortality districts, may be a starting point for quality improvement efforts. The exploration of associations at lower and higher thresholds of quality provides initial evidence that quality improvements are needed at most facilities; targeting only the lowest-performing facilities is unlikely to affect mortality. However, evidence for interventions that can rapidly improve quality of delivery care at scale is limited to date [41]. Given that larger facilities and hospitals had better quality performance, one strategy for providing women with better care is regionalizing delivery care to highest-quality centers while improving transport for women to reach these facilities [42].

Beyond Malawi, these results argue for pivoting from a focus on access to delivery facilities to measuring and improving quality of these facilities in the pursuit of reduced neonatal mortality. Although access to care is essential, ambitious global targets for newborn and child survival can be met only if the care that women receive is of sufficient quality.

## Supporting Information

S1 Fig Causal model of delivery quality and neonatal mortality. (TIF)

S1 STROBE Checklist (DOC)

S1 Table Instrument strength across analytic models. (XLSX)

S2 Table Distribution of potential confounders by categories of differential distance. (XLSX)

S3 Table Falsification tests of differential distance and neonatal mortality. (XLSX)

S4 Table Bounds on IV estimate. (XLSX)

S5 Table IV analysis of coarse quality indicators and neonatal mortality. (XLSX)

S6 Table IV analysis of range of thresholds for high-quality facility *(n =* 6,668). (XLSX)

S1 Text Conceptual framework for IV approach. (DOCX)

S2 Text Testing of assumptions required for consistent estimation of IV model. (DOCX)

S3 Text Interpreting LATE size. (DOCX)

S4 Text Full IV regression results in linear probability and probit models. (DOCX)

## Abbreviations

ANC: Antenatal care
BCG: Bacille Calmette-Guérin vaccine
CHAM: Christian Health Association of Malawi
CI: Confidence interval
EA: Enumeration area
IV: Instrumental variable
LATE: Local average treatment effect
LBW: low birth weight
LMIC: low- and middle-income countries
MDG: Millennium Development Goals
MES: Millennium Development Goals Endline Survey
MICS: Multiple indicator cluster survey
PCA: Principal components analysis
SD: Standard deviation
SPA: Service Provision Assessment
UNICEF: United Nations Children’s Fund
WHO: World Health Organization
