# Supplementary figures and images for: Obstetric Facility Quality and Newborn Mortality in Malawi: A Cross-Sectional Study
Source: PLoS Med. 2016 Oct 18;13(10):e1002151. doi: 10.1371/journal.pmed.1002151 (PMC5068819; doi:10.1371/journal.pmed.1002151)

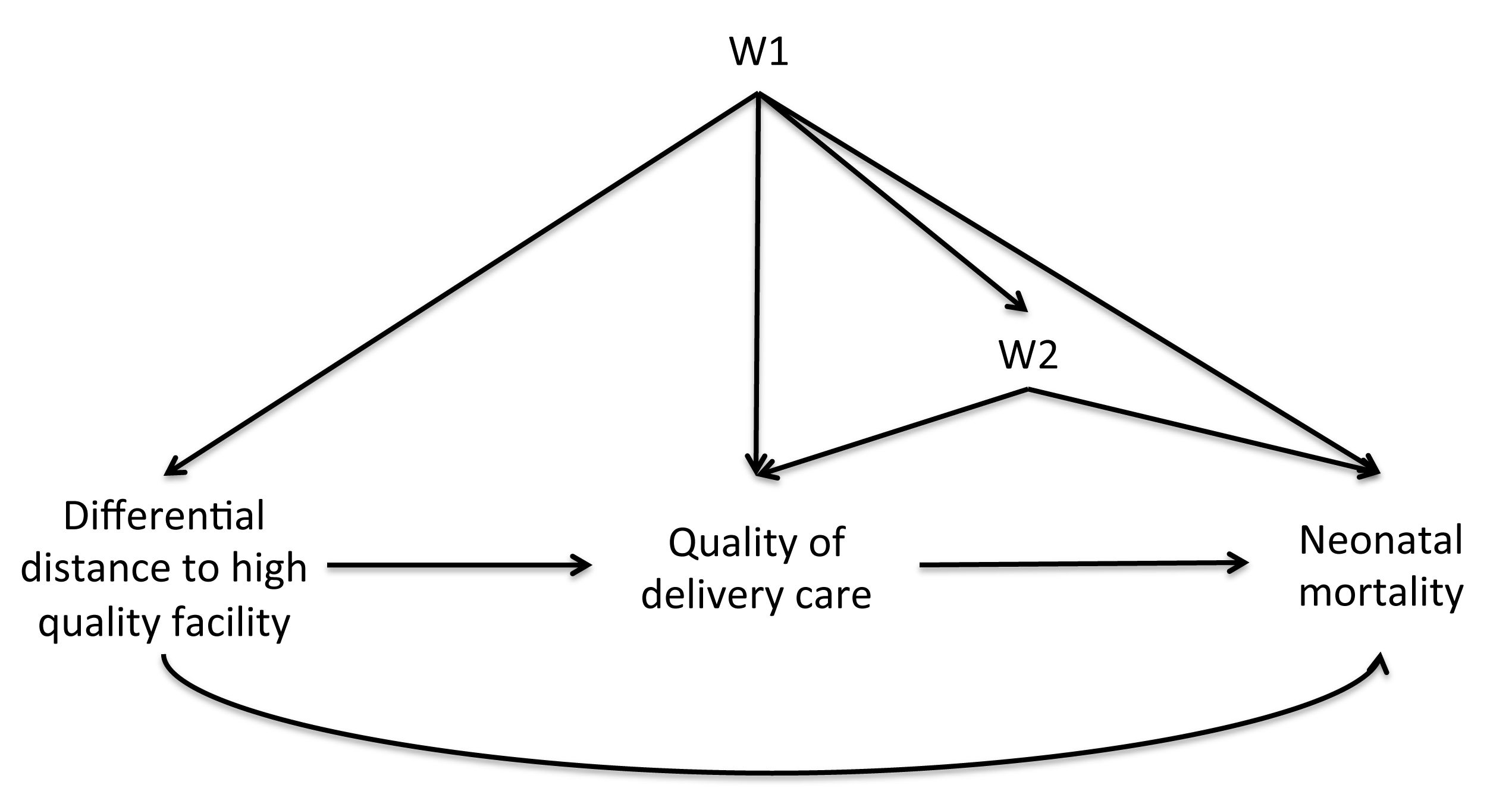

Supplement: S1 Fig — (TIF) [file pmed.1002151.s001.tif]
