# Supplementary material for: Obstetric Facility Quality and Newborn Mortality in Malawi: A Cross-Sectional Study
Source: PLoS Med. 2016 Oct 18;13(10):e1002151. doi: 10.1371/journal.pmed.1002151 (PMC5068819; doi:10.1371/journal.pmed.1002151)
Supplement: S1 Text — (DOCX) [file pmed.1002151.s009.docx]

**S1 Text. Conceptual framework for instrumental variable approach**

S1 Fig provides an overall causal model for the relationship of the quality of delivery care (exposure) with neonatal mortality (outcome). The difference in distance to a high quality facility versus distance to the nearest delivery facility is employed as an instrumental variable (IV). W1 represents all common causes of differential distance to a high quality facility and neonatal mortality, both measured and unmeasured. These would include the quality and density of the local health system as well as any contextual factors, such as urban vs. rural area, that could affect the IV and the outcome. These factors may also influence delivery care quality at the facility where women deliver and the set of variables in W2. W2 includes all common causes of quality of delivery care received and neonatal mortality. It includes selection preferences by individual women into specific facilities, pre-labor risks leading to referral prior to delivery, and complications during labor that lead to transfer to higher-level facilities. Identification of risks before and during delivery and appropriate referral are affected by W1 factors such as local health system quality.

Based on the conceptual model in S1 Fig, the W1 covariates selected for the IV model were: urban location and density of local health system (logged number of health facilities within 20 kilometers). We additionally controlled for W2 covariates (maternal educational status, maternal household wealth quintile, maternal age <18 at delivery, multiple births, primiparity, male infant, and low birthweight infant) to increase precision in estimation.[1]

1. Pizer SD. Falsification Testing of Instrumental Variables Methods for Comparative Effectiveness Research. Health Serv Res. 2016;51(2):790-811. Epub 2015/08/22. doi: 10.1111/1475-6773.12355. PubMed PMID: 26293167; PubMed Central PMCID: PMCPMC4799892.
