# Supplementary material for: Obstetric Facility Quality and Newborn Mortality in Malawi: A Cross-Sectional Study
Source: PLoS Med. 2016 Oct 18;13(10):e1002151. doi: 10.1371/journal.pmed.1002151 (PMC5068819; doi:10.1371/journal.pmed.1002151)
Supplement: S2 Text — (DOCX) [file pmed.1002151.s010.docx]

**S2 Text. Testing of assumptions required for consistent estimation of IV model**

Validity of the instrumental variables estimates is contingent on the following assumptions, stated generally and applied to this analysis. We discuss each assumption in turn.

1. The instrument is associated with the exposure: differential distance to a high quality facility should be related to a woman’s probability of delivering in a high quality facility.

This assumption is satisfied: relative accessibility of high quality facility strongly affects probability of delivering in a high quality facility, with 75.4% of women whose closest facility is high quality delivering at a high quality facility compared to 7.1% of women who are more than 30 kilometers further from high quality care than any care. The F tests reported in the main analysis and repeated below show robust instrument strength in all model specifications, adjusting for all covariates. (An F test of 30 is considered to represent adequate strength of the first stage)

1. Exclusion restriction: the instrument does not affect the outcome except through treatment, i.e. the direct path from differential distance to neonatal mortality can be excluded from the causal diagram. Aside from the quality of the care at the facility where a woman delivers, the relative accessibility of high quality delivery care should not affect risk of neonatal death.

This assumption is not directly verifiable; we assess it using subject-matter knowledge and we attempt two falsification tests for assumptions 2 and 3, reported subsequently.

We argue that differential distance meets the exclusion restriction, as we are unable to identify plausible causal links from differential distance from nearest to high quality facility to mortality that is not mediated by quality of delivery facility. It is highly plausible that differential distance between nearest and higher quality facility is randomly distributed with respect to risk of newborn mortality. Facilities are placed geographically to achieve population coverage; the quality of inputs and processes in facilities is determined by proximity to urban areas, management, and supply chain effectiveness, and is unlikely to be a direct cause of mortality risk. While it is possible that women experiencing complications would not be transferred at all or in time to referral facilities if the distance is too great, increasing risk of neonatal death, this pathway still depends on the quality of the initial admitting facility being low.

1. The instrument does not share any causes with the outcome: there are no common factors affecting both differential distance and neonatal mortality.

As with assumption 2, it is not possible to directly verify this assumption. We assert that differential distance and neonatal mortality are independent conditional on the common causes of urban location and density of the local health system, i.e. that these identified covariates are part of the causal pathway from any common cause to IV and outcome. As support of this claim, we assessed the distribution of known confounders by strata of the instrument, separately for urban and rural populations. Even distribution of confounders would suggest the instrument is effective in pseud-randomizing the population to high or lower quality delivery care. As shown in S2 Table, most confounding variables were evenly distributed by categories of the instrument (using quartiles for rural women and tertiles for urban women given the much smaller range of distance in urban women). This table does not reflect the additional adjustment for the proposed common cause of health system density in the analytic model, but lends credibility to assumption 3 conditioning on urban location alone.

We conducted two falsification tests of the IV.[1] First, we assessed the relationship between differential distance and mortality among births that took place outside of a health facility. A strong relationship between IV and outcome in a population that is not exposed to treatment (delivery in a higher quality facility) falsifies assumptions 2 and 3. Second, we assessed the relationship between differential distance and diarrhea in the past two weeks in any of the study mothers’ children under 5. There should not be a causal relationship between intrapartum care and childhood illness, but child illness could be affected by contextual factors that may confound our main analysis, such as health system inputs. A strong relationship between the IV and this alternative outcome indicates uncontrolled pathways connecting the instrument and mortality.

For both tests we performed linear regression of death on the IV, weighted with women’s sampling weight. We controlled for all covariates in test model 1 and contextual and maternal covariates in test model 2. S3 Table shows the association between differential distance and infant mortality in these two specifications.

Neither test shows evidence of a strong relationship between differential distance and the outcome; the IV is not rejected based on these tests.

We proceed with differential distance as a plausibly valid instrument based on the evidence above.

1. Pizer SD. Falsification Testing of Instrumental Variables Methods for Comparative Effectiveness Research. Health Serv Res. 2016;51(2):790-811. Epub 2015/08/22. doi: 10.1111/1475-6773.12355. PubMed PMID: 26293167; PubMed Central PMCID: PMCPMC4799892.
