# Supplementary material for: Obstetric Facility Quality and Newborn Mortality in Malawi: A Cross-Sectional Study
Source: PLoS Med. 2016 Oct 18;13(10):e1002151. doi: 10.1371/journal.pmed.1002151 (PMC5068819; doi:10.1371/journal.pmed.1002151)
Supplement: S3 Text — (DOCX) [file pmed.1002151.s011.docx]

**S3 Text. Interpreting local average treatment effect size**

The effect of interest in this analysis is the local average treatment effect (LATE), the estimated effect of delivering at a higher quality facility among women who would deliver at such a facility if one were more readily accessible but would not deliver at such a facility otherwise (termed compliers). In this context, that might exclude extremely high-risk women who will always deliver at high quality facilities or women with very strong preferences for a specific type of facility, regardless of quality. The observed data suggest a large portion of the population may be compliers, given the dramatic increase in probability of delivering at a high quality facility as relative distance to such a facility decreases (<10% for women beyond 30 kilometers differential distance to >75% for women whose nearest facility is high quality).

The LATE requires one additional assumption: monotonicity, or no “defiers” or “never takers”- women who would only deliver in a high quality facility were it relatively further from them.(2) Although this assumption is not empirically verifiable, we find no conceptual reason to believe such women would exist. While women may have strong preferences for delivery location, including home births, invoking such preferences only if a high quality facility were nearby does not appear credible.

The monotonicity assumption is not required to estimate bounds on the instrumental variable estimate. We therefore employed a categorical version of the instrument in order to calculate bounds on an unadjusted IV estimate. We categorized differential distance using the categories in S2 Table above, collapsing the middle two quartiles of distance in rural areas. We present these bounds in S4 Table; the breadth of the estimates indicates the importance of the monotonicity assumption to the validity of our point estimate.
