# Supplementary material for: Obstetric Facility Quality and Newborn Mortality in Malawi: A Cross-Sectional Study
Source: PLoS Med. 2016 Oct 18;13(10):e1002151. doi: 10.1371/journal.pmed.1002151 (PMC5068819; doi:10.1371/journal.pmed.1002151)
Supplement: S4 Text — (DOCX) [file pmed.1002151.s012.docx]

**S4 Text. Full IV regression results in linear probability and probit models**

*Main IV model

*Exposure: Delivery at high quality facility (top 25% of all delivery facilities)

*IV: Difference between distance to nearest delivery facility and nearest high quality facility

svy: ivregress 2sls death28 urban cluster_facrad20kmlog i.hh_windex5 w_edsec w_lt18 inf_male inf_mult inf_primi inf_lbw (q_indexbestf1 = iv_difdistbest)

(running ivregress on estimation sample)

Survey: Instrumental variables (2SLS) regression

Number of strata = 55 Number of obs = 6,668

Number of PSUs = 1,120 Population size = 6,668

Design df = 1,065

F( 13, 1053) = 3.92

Prob > F = 0.0000

R-squared = 0.0163

---------------------------------------------------------------------------------------

| Linearized

death28days | Coef. Std. Err. t P>|t| [95% Conf. Interval]

----------------------+----------------------------------------------------------------

q_indexbestf1 | -.0231258 .0116073 -1.99 0.047 -.0459016 -.00035

urban | .0040642 .0069713 0.58 0.560 -.0096149 .0177433

cluster_facrad20kmlog | -.0019722 .002648 -0.74 0.457 -.0071681 .0032237

|

hh_windex5 |

Second | -.0014869 .0057198 -0.26 0.795 -.0127101 .0097364

Middle | -.0008435 .0056685 -0.15 0.882 -.0119661 .0102792

Fourth | -.0089881 .0049708 -1.81 0.071 -.0187417 .0007654

Richest | .0039487 .0069788 0.57 0.572 -.0097451 .0176425

|

w_edsec | -.0072189 .0048739 -1.48 0.139 -.0167824 .0023447

w_lt18birth | .0026703 .0065895 0.41 0.685 -.0102597 .0156002

inf_male | .0085248 .003169 2.69 0.007 .0023065 .0147431

inf_multiple | .0891177 .0218477 4.08 0.000 .0462482 .1319871

inf_primip | .0162605 .0053889 3.02 0.003 .0056864 .0268347

inf_lbw | .0138534 .0062948 2.20 0.028 .0015018 .0262049

_cons | .0208305 .01001 2.08 0.038 .001189 .0404721

---------------------------------------------------------------------------------------

Instrumented: q_indexbestf1

Instruments: urban cluster_facrad20kmlog 2.hh_windex5 3.hh_windex5 4.hh_windex5

5.hh_windex5 w_edsec w_lt18birth inf_male inf_multiple inf_primip

inf_lbw iv_difdistbest

---------------------------------------------------------------------------------------

svy: ivprobit death28 urban cluster_facrad20kmlog i.hh_windex5 w_edsec w_lt18 inf_male inf_mult inf_primi inf_lbw (q_indexbestf1 = iv_difdistbest)

(running ivprobit on estimation sample)

Survey: Probit model with endogenous regressors

Number of strata = 55 Number of obs = 6,668

Number of PSUs = 1,120 Population size = 6,668

Design df = 1,065

F( 13, 1053) = 9.15

Prob > F = 0.0000

---------------------------------------------------------------------------------------

| Linearized

| Coef. Std. Err. t P>|t| [95% Conf. Interval]

----------------------+----------------------------------------------------------------

death28days |

q_indexbestf1 | -.4012759 .1781625 -2.25 0.025 -.7508652 -.0516866

urban | .0520662 .1617199 0.32 0.748 -.2652596 .3693919

cluster_facrad20kmlog | -.0552072 .0576581 -0.96 0.339 -.1683436 .0579292

|

hh_windex5 |

Second | -.0099353 .117481 -0.08 0.933 -.2404558 .2205853

Middle | -.0366241 .1187649 -0.31 0.758 -.2696638 .1964156

Fourth | -.2953404 .1420165 -2.08 0.038 -.5740044 -.0166764

Richest | .0952278 .1490456 0.64 0.523 -.1972286 .3876842

|

w_edsec | -.1947766 .1286134 -1.51 0.130 -.4471411 .0575879

w_lt18birth | .046481 .1109053 0.42 0.675 -.1711367 .2640988

inf_male | .1893368 .0756157 2.50 0.012 .0409642 .3377094

inf_multiple | .9655729 .1289853 7.49 0.000 .7124787 1.218667

inf_primip | .3665734 .1004086 3.65 0.000 .1695523 .5635946

inf_lbw | .2484552 .093855 2.65 0.008 .0642934 .432617

_cons | -2.043 .1986664 -10.28 0.000 -2.432822 -1.653178

----------------------+----------------------------------------------------------------

q_indexbestf1 |

urban | .1921104 .0284994 6.74 0.000 .136189 .2480318

cluster_facrad20kmlog | -.0469169 .014963 -3.14 0.002 -.0762772 -.0175565

|

hh_windex5 |

Second | -.0343681 .0170794 -2.01 0.044 -.0678811 -.000855

Middle | -.0001234 .0172772 -0.01 0.994 -.0340247 .0337779

Fourth | .0084614 .0203388 0.42 0.677 -.0314473 .04837

Richest | .022613 .0263921 0.86 0.392 -.0291735 .0743995

|

w_edsec | .028474 .0165276 1.72 0.085 -.0039564 .0609044

w_lt18birth | .0083905 .0210713 0.40 0.691 -.0329554 .0497364

inf_male | -.0071767 .011303 -0.63 0.526 -.0293554 .015002

inf_multiple | .13362 .0387374 3.45 0.001 .0576097 .2096303

inf_primip | .0550579 .0166096 3.31 0.001 .0224666 .0876492

inf_lbw | .0102334 .0157863 0.65 0.517 -.0207423 .0412091

iv_difdistbest | -.021824 .0012453 -17.53 0.000 -.0242675 -.0193805

_cons | .6795987 .0450863 15.07 0.000 .5911307 .7680667

----------------------+----------------------------------------------------------------

/athrho2_1 | .2805316 .0959913 2.92 0.004 .0921782 .4688851

/lnsigma2 | -.8033525 .0069732 -115.21 0.000 -.8170353 -.7896696

----------------------+----------------------------------------------------------------

corr(e.q_indexbestf1,|

e.death28days)| .2733971 .0888163 .091918 .4372981

sd(e.q_indexbestf1)| .4478251 .0031228 .4417393 .4539947

---------------------------------------------------------------------------------------

Instrumented: q_indexbestf1

Instruments: urban cluster_facrad20kmlog 2.hh_windex5 3.hh_windex5 4.hh_windex5

5.hh_windex5 w_edsec w_lt18birth inf_male inf_multiple inf_primip

inf_lbw iv_difdistbest

---------------------------------------------------------------------------------------

*Robustness check 1: controlling for hospital

svy: ivregress 2sls death28 urban cluster_facrad20kmlog i.hh_windex5 w_edsec w_lt18 inf_male inf_mult inf_primi inf_lbw del_hosp (q_indexbestf1 = iv_difdistbest)

(running ivregress on estimation sample)

Survey: Instrumental variables (2SLS) regression

Number of strata = 55 Number of obs = 6,668

Number of PSUs = 1,120 Population size = 6,668

Design df = 1,065

F( 14, 1052) = 3.70

Prob > F = 0.0000

R-squared = 0.0170

---------------------------------------------------------------------------------------

| Linearized

death28days | Coef. Std. Err. t P>|t| [95% Conf. Interval]

----------------------+----------------------------------------------------------------

q_indexbestf1 | -.0425278 .0189165 -2.25 0.025 -.0796455 -.00541

urban | -.0024282 .0060747 -0.40 0.689 -.0143478 .0094915

cluster_facrad20kmlog | -.001439 .0026822 -0.54 0.592 -.0067019 .003824

|

hh_windex5 |

Second | -.0019512 .0057085 -0.34 0.733 -.0131525 .0092501

Middle | -.0021098 .0057741 -0.37 0.715 -.0134397 .0092201

Fourth | -.0110708 .0051138 -2.16 0.031 -.0211051 -.0010366

Richest | -.0003408 .0071755 -0.05 0.962 -.0144205 .0137389

|

w_edsec | -.0081859 .0048849 -1.68 0.094 -.017771 .0013992

w_lt18birth | .0024266 .006602 0.37 0.713 -.0105279 .0153811

inf_male | .0080443 .003176 2.53 0.011 .0018124 .0142762

inf_multiple | .0836558 .0215409 3.88 0.000 .0413883 .1259232

inf_primip | .0138695 .0052635 2.64 0.009 .0035415 .0241975

inf_lbw | .0137983 .0062849 2.20 0.028 .0014661 .0261304

del_hospital | .0402688 .0153772 2.62 0.009 .0100958 .0704418

_cons | .0158301 .0092912 1.70 0.089 -.002401 .0340613

---------------------------------------------------------------------------------------

Instrumented: q_indexbestf1

Instruments: urban cluster_facrad20kmlog 2.hh_windex5 3.hh_windex5 4.hh_windex5

5.hh_windex5 w_edsec w_lt18birth inf_male inf_multiple inf_primip

inf_lbw del_hospital iv_difdistbest

---------------------------------------------------------------------------------------

svy: ivprobit death28 urban cluster_facrad20kmlog i.hh_windex5 w_edsec w_lt18 inf_male inf_mult inf_primi inf_lbw del_hosp (q_indexbestf1 = iv_difdistbest)

(running ivprobit on estimation sample)

Survey: Probit model with endogenous regressors

Number of strata = 55 Number of obs = 6,668

Number of PSUs = 1,120 Population size = 6,668

Design df = 1,065

F( 14, 1052) = 8.69

Prob > F = 0.0000

---------------------------------------------------------------------------------------

| Linearized

| Coef. Std. Err. t P>|t| [95% Conf. Interval]

----------------------+----------------------------------------------------------------

death28days |

q_indexbestf1 | -.7751643 .2925083 -2.65 0.008 -1.349122 -.2012064

urban | -.0678991 .1484534 -0.46 0.647 -.3591935 .2233952

cluster_facrad20kmlog | -.0478484 .0594915 -0.80 0.421 -.1645823 .0688854

|

hh_windex5 |

Second | -.0220987 .1170306 -0.19 0.850 -.2517353 .207538

Middle | -.0649854 .1212294 -0.54 0.592 -.302861 .1728902

Fourth | -.3367349 .1439192 -2.34 0.019 -.6191323 -.0543376

Richest | .0046986 .1501577 0.03 0.975 -.28994 .2993371

|

w_edsec | -.2149663 .1295697 -1.66 0.097 -.4692073 .0392746

w_lt18birth | .0357823 .1125316 0.32 0.751 -.1850265 .2565911

inf_male | .1804683 .0763815 2.36 0.018 .0305929 .3303436

inf_multiple | .8635657 .131551 6.56 0.000 .6054371 1.121694

inf_primip | .3244592 .1014442 3.20 0.001 .125406 .5235124

inf_lbw | .2522384 .093428 2.70 0.007 .0689145 .4355623

del_hospital | .7773924 .2416589 3.22 0.001 .3032108 1.251574

_cons | -2.141731 .1930983 -11.09 0.000 -2.520627 -1.762835

----------------------+----------------------------------------------------------------

q_indexbestf1 |

urban | .0100478 .0184627 0.54 0.586 -.0261797 .0462752

cluster_facrad20kmlog | -.0212005 .0096306 -2.20 0.028 -.0400976 -.0023034

|

hh_windex5 |

Second | -.0307606 .0116396 -2.64 0.008 -.0535996 -.0079215

Middle | -.0226405 .0125661 -1.80 0.072 -.0472976 .0020167

Fourth | -.0315661 .0137295 -2.30 0.022 -.0585061 -.0046261

Richest | -.0616195 .0153864 -4.00 0.000 -.0918105 -.0314285

|

w_edsec | .0014046 .0112916 0.12 0.901 -.0207517 .0235609

w_lt18birth | .0011493 .014871 0.08 0.938 -.0280305 .0303291

inf_male | -.0132562 .007629 -1.74 0.083 -.0282259 .0017134

inf_multiple | -.0098682 .0223864 -0.44 0.659 -.0537948 .0340583

inf_primip | -.0065688 .0111812 -0.59 0.557 -.0285086 .0153709

inf_lbw | .0057142 .0104357 0.55 0.584 -.0147626 .0261911

del_hospital | .7173859 .0156907 45.72 0.000 .6865978 .748174

iv_difdistbest | -.0142807 .0010511 -13.59 0.000 -.0163431 -.0122182

_cons | .3556174 .0322165 11.04 0.000 .2924023 .4188325

----------------------+----------------------------------------------------------------

/athrho2_1 | .2669894 .1046757 2.55 0.011 .0615953 .4723836

/lnsigma2 | -1.192281 .0230834 -51.65 0.000 -1.237576 -1.146987

----------------------+----------------------------------------------------------------

corr(e.q_indexbestf1,|

e.death28days)| .2608213 .0975549 .0615175 .4401232

sd(e.q_indexbestf1)| .303528 .0070065 .2900866 .3175922

---------------------------------------------------------------------------------------

Instrumented: q_indexbestf1

Instruments: urban cluster_facrad20kmlog 2.hh_windex5 3.hh_windex5 4.hh_windex5

5.hh_windex5 w_edsec w_lt18birth inf_male inf_multiple inf_primip

inf_lbw del_hospital iv_difdistbest

---------------------------------------------------------------------------------------

*Robustness check 2: higher quality threshold, absolute cut point

*Exposure: Delivery at high quality facility ( > 0.80 on quality index)

*IV: Difference between distance to nearest delivery facility and nearest high quality (> 0.80) facility

svy: ivregress 2sls death28 urban cluster_facrad20kmlog i.hh_windex5 w_edsec w_lt18 inf_male inf_mult inf_primi inf_lbw (q_indexgte80f1 = iv_difdistgte80)

(running ivregress on estimation sample)

Survey: Instrumental variables (2SLS) regression

Number of strata = 55 Number of obs = 6,668

Number of PSUs = 1,120 Population size = 6,668

Design df = 1,065

F( 13, 1053) = 3.98

Prob > F = 0.0000

R-squared = 0.0150

---------------------------------------------------------------------------------------

| Linearized

death28days | Coef. Std. Err. t P>|t| [95% Conf. Interval]

----------------------+----------------------------------------------------------------

q_indexgte80f1 | -.0302536 .0140746 -2.15 0.032 -.0578707 -.0026365

urban | .0069765 .0075594 0.92 0.356 -.0078565 .0218094

cluster_facrad20kmlog | -.0024956 .0026901 -0.93 0.354 -.0077742 .002783

|

hh_windex5 |

Second | -.0012398 .005709 -0.22 0.828 -.012442 .0099624

Middle | -.0006933 .0056454 -0.12 0.902 -.0117706 .010384

Fourth | -.0079119 .0050164 -1.58 0.115 -.0177551 .0019312

Richest | .0050985 .0069684 0.73 0.465 -.0085749 .0187719

|

w_edsec | -.0073518 .0048738 -1.51 0.132 -.0169151 .0022115

w_lt18birth | .0020387 .0066829 0.31 0.760 -.0110744 .0151517

inf_male | .0088325 .0031726 2.78 0.005 .0026073 .0150577

inf_multiple | .0908142 .0220381 4.12 0.000 .0475713 .1340572

inf_primip | .0173185 .0055957 3.09 0.002 .0063387 .0282983

inf_lbw | .0140679 .006317 2.23 0.026 .0016727 .0264631

_cons | .020975 .0098798 2.12 0.034 .0015889 .040361

---------------------------------------------------------------------------------------

Instrumented: q_indexgte80f1

Instruments: urban cluster_facrad20kmlog 2.hh_windex5 3.hh_windex5 4.hh_windex5

5.hh_windex5 w_edsec w_lt18birth inf_male inf_multiple inf_primip

inf_lbw iv_difdistgte80

---------------------------------------------------------------------------------------

svy: ivprobit death28 urban cluster_facrad20kmlog i.hh_windex5 w_edsec w_lt18 inf_male inf_mult inf_primi inf_lbw (q_indexgte80f1 = iv_difdistgte80)

(running ivprobit on estimation sample)

Survey: Probit model with endogenous regressors

Number of strata = 55 Number of obs = 6,668

Number of PSUs = 1,120 Population size = 6,668

Design df = 1,065

F( 13, 1053) = 9.23

Prob > F = 0.0000

----------------------------------------------------------------------------------------

| Linearized

| Coef. Std. Err. t P>|t| [95% Conf. Interval]

-----------------------+----------------------------------------------------------------

death28days |

q_indexgte80f1 | -.5353553 .2114582 -2.53 0.011 -.9502772 -.1204334

urban | .0943216 .1671035 0.56 0.573 -.233568 .4222111

cluster_facrad20kmlog | -.0632843 .0578069 -1.09 0.274 -.1767127 .050144

|

hh_windex5 |

Second | .0011377 .1172218 0.01 0.992 -.2288741 .2311496

Middle | -.0252481 .1184409 -0.21 0.831 -.2576522 .207156

Fourth | -.2790506 .1420274 -1.96 0.050 -.5577359 -.0003653

Richest | .1160354 .1492884 0.78 0.437 -.1768975 .4089683

|

w_edsec | -.1875514 .1286898 -1.46 0.145 -.4400656 .0649629

w_lt18birth | .0417768 .1109427 0.38 0.707 -.1759144 .2594679

inf_male | .1926461 .0756689 2.55 0.011 .044169 .3411232

inf_multiple | .9933262 .1315662 7.55 0.000 .7351679 1.251485

inf_primip | .3845938 .1001629 3.84 0.000 .1880548 .5811328

inf_lbw | .2451663 .0942138 2.60 0.009 .0603006 .430032

_cons | -2.036139 .2005202 -10.15 0.000 -2.429598 -1.64268

-----------------------+----------------------------------------------------------------

q_indexgte80f1 |

urban | .2301718 .0278594 8.26 0.000 .1755063 .2848373

cluster_facrad20kmlog | -.0623935 .0133462 -4.68 0.000 -.0885813 -.0362058

|

hh_windex5 |

Second | -.0184531 .0162659 -1.13 0.257 -.05037 .0134638

Middle | -.0056995 .015895 -0.36 0.720 -.0368885 .0254896

Fourth | .0235248 .0197772 1.19 0.235 -.015282 .0623315

Richest | .0381652 .0253664 1.50 0.133 -.0116087 .087939

|

w_edsec | .01501 .0161206 0.93 0.352 -.0166217 .0466418

w_lt18birth | -.0046277 .0203747 -0.23 0.820 -.0446067 .0353513

inf_male | .0035572 .0105676 0.34 0.736 -.0171784 .0242929

inf_multiple | .1486442 .0365633 4.07 0.000 .0768999 .2203885

inf_primip | .0662035 .0164255 4.03 0.000 .0339735 .0984334

inf_lbw | .0135927 .0153925 0.88 0.377 -.0166104 .0437957

iv_difdistgte80 | -.0138078 .0007128 -19.37 0.000 -.0152065 -.0124092

_cons | .601783 .0411048 14.64 0.000 .5211274 .6824386

-----------------------+----------------------------------------------------------------

/athrho2_1 | .2806645 .107341 2.61 0.009 .0700407 .4912884

/lnsigma2 | -.8459459 .0086394 -97.92 0.000 -.862898 -.8289938

-----------------------+----------------------------------------------------------------

corr(e.q_indexgte80f1,|

e.death28days)| .27352 .0993105 .0699264 .4552384

sd(e.q_indexgte80f1)| .4291512 .0037076 .4219375 .4364882

----------------------------------------------------------------------------------------

Instrumented: q_indexgte80f1

Instruments: urban cluster_facrad20kmlog 2.hh_windex5 3.hh_windex5 4.hh_windex5

5.hh_windex5 w_edsec w_lt18birth inf_male inf_multiple inf_primip

inf_lbw iv_difdistgte80

----------------------------------------------------------------------------------------

*Robustness check 3: lower quality threshold

*Exposure: top 33% of all delivery facilities

*IV: Difference between distance to nearest delivery facility and nearest high quality facility (top 33%)

svy: ivregress 2sls death28 urban cluster_facrad20kmlog i.hh_windex5 w_edsec w_lt18 inf_male inf_mult inf_primi inf_lbw (q_index33f1 = iv_difdist33)

(running ivregress on estimation sample)

Survey: Instrumental variables (2SLS) regression

Number of strata = 55 Number of obs = 6,668

Number of PSUs = 1,120 Population size = 6,668

Design df = 1,065

F( 13, 1053) = 3.94

Prob > F = 0.0000

R-squared = 0.0093

---------------------------------------------------------------------------------------

| Linearized

death28days | Coef. Std. Err. t P>|t| [95% Conf. Interval]

----------------------+----------------------------------------------------------------

q_index33f1 | -.03274 .0129596 -2.53 0.012 -.0581692 -.0073108

urban | .0052602 .0067564 0.78 0.436 -.0079971 .0185176

cluster_facrad20kmlog | -.0006642 .0025986 -0.26 0.798 -.0057632 .0044348

|

hh_windex5 |

Second | -.0016311 .0057551 -0.28 0.777 -.0129238 .0096616

Middle | -.0004081 .0056603 -0.07 0.943 -.0115146 .0106985

Fourth | -.0079043 .0050144 -1.58 0.115 -.0177436 .0019349

Richest | .0051787 .0069573 0.74 0.457 -.0084728 .0188303

|

w_edsec | -.0073641 .0048972 -1.50 0.133 -.0169733 .0022451

w_lt18birth | .0031578 .0065945 0.48 0.632 -.009782 .0160975

inf_male | .0085774 .0031861 2.69 0.007 .0023256 .0148291

inf_multiple | .0903077 .0218811 4.13 0.000 .0473727 .1332427

inf_primip | .0164755 .0054143 3.04 0.002 .0058516 .0270993

inf_lbw | .0137548 .0063087 2.18 0.029 .0013759 .0261337

_cons | .0235358 .0103896 2.27 0.024 .0031495 .0439221

---------------------------------------------------------------------------------------

Instrumented: q_index33f1

Instruments: urban cluster_facrad20kmlog 2.hh_windex5 3.hh_windex5 4.hh_windex5

5.hh_windex5 w_edsec w_lt18birth inf_male inf_multiple inf_primip

inf_lbw iv_difdist33

---------------------------------------------------------------------------------------

svy: ivprobit death28 urban cluster_facrad20kmlog i.hh_windex5 w_edsec w_lt18 inf_male inf_mult inf_primi inf_lbw (q_index33f1 = iv_difdist33)

(running ivprobit on estimation sample)

Survey: Probit model with endogenous regressors

Number of strata = 55 Number of obs = 6,668

Number of PSUs = 1,120 Population size = 6,668

Design df = 1,065

F( 13, 1053) = 9.85

Prob > F = 0.0000

---------------------------------------------------------------------------------------

| Linearized

| Coef. Std. Err. t P>|t| [95% Conf. Interval]

----------------------+----------------------------------------------------------------

death28days |

q_index33f1 | -.5188393 .1748704 -2.97 0.003 -.861969 -.1757096

urban | .0635855 .1553308 0.41 0.682 -.2412036 .3683745

cluster_facrad20kmlog | -.0224488 .056948 -0.39 0.694 -.1341918 .0892941

|

hh_windex5 |

Second | -.0120787 .1161812 -0.10 0.917 -.2400488 .2158914

Middle | -.0279683 .1168205 -0.24 0.811 -.2571928 .2012561

Fourth | -.2833142 .141908 -2.00 0.046 -.5617653 -.0048631

Richest | .1103344 .1467711 0.75 0.452 -.1776589 .3983277

|

w_edsec | -.1918588 .1261646 -1.52 0.129 -.4394183 .0557006

w_lt18birth | .0595671 .108988 0.55 0.585 -.1542884 .2734227

inf_male | .1847642 .0752505 2.46 0.014 .0371082 .3324203

inf_multiple | .9636163 .1259192 7.65 0.000 .7165384 1.210694

inf_primip | .3586642 .0984013 3.64 0.000 .1655817 .5517468

inf_lbw | .2449808 .0925237 2.65 0.008 .0634314 .4265301

_cons | -2.01133 .1911594 -10.52 0.000 -2.386421 -1.636238

----------------------+----------------------------------------------------------------

q_index33f1 |

urban | .1578374 .0281819 5.60 0.000 .1025391 .2131357

cluster_facrad20kmlog | -.0306076 .0154275 -1.98 0.048 -.0608793 -.000336

|

hh_windex5 |

Second | -.0282856 .017368 -1.63 0.104 -.0623649 .0057938

Middle | .0107491 .0175552 0.61 0.540 -.0236975 .0451958

Fourth | .0280832 .0197585 1.42 0.156 -.0106868 .0668531

Richest | .0428018 .0260291 1.64 0.100 -.0082723 .0938759

|

w_edsec | .0192189 .0163279 1.18 0.239 -.0128197 .0512575

w_lt18birth | .0120442 .021296 0.57 0.572 -.0297427 .0538311

inf_male | -.0017304 .0110798 -0.16 0.876 -.0234712 .0200103

inf_multiple | .1327477 .0371835 3.57 0.000 .0597864 .205709

inf_primip | .0490201 .0168212 2.91 0.004 .0160136 .0820265

inf_lbw | .0096115 .0157502 0.61 0.542 -.0212934 .0405164

iv_difdist33 | -.0273315 .0015432 -17.71 0.000 -.0303596 -.0243033

_cons | .6983946 .0461171 15.14 0.000 .6079039 .7888852

----------------------+----------------------------------------------------------------

/athrho2_1 | .3307531 .0940527 3.52 0.000 .1462034 .5153027

/lnsigma2 | -.8071234 .0082828 -97.45 0.000 -.8233758 -.790871

----------------------+----------------------------------------------------------------

corr(e.q_index33f1,|

e.death28days)| .3191973 .08447 .1451705 .4740665

sd(e.q_index33f1)| .4461396 .0036953 .4389474 .4534497

---------------------------------------------------------------------------------------

Instrumented: q_index33f1

Instruments: urban cluster_facrad20kmlog 2.hh_windex5 3.hh_windex5 4.hh_windex5

5.hh_windex5 w_edsec w_lt18birth inf_male inf_multiple inf_primip

inf_lbw iv_difdist33

---------------------------------------------------------------------------------------

*Robustness check 4: PCA-based metric of quality

*Exposure: Top 25% of all delivery facilities based on first component of PCA of 25 quality items

*IV: difference between distance to nearest delivery facility and nearest high quality facility

svy: ivregress 2sls death28 urban cluster_facrad20kmlog i.hh_windex5 w_edsec w_lt18 inf_male inf_mult inf_primi inf_lbw (q_pca1bestf1 = iv_difdistpca)

(running ivregress on estimation sample)

Survey: Instrumental variables (2SLS) regression

Number of strata = 55 Number of obs = 6,668

Number of PSUs = 1,120 Population size = 6,668

Design df = 1,065

F( 13, 1053) = 3.91

Prob > F = 0.0000

R-squared = 0.0136

---------------------------------------------------------------------------------------

| Linearized

death28days | Coef. Std. Err. t P>|t| [95% Conf. Interval]

----------------------+----------------------------------------------------------------

q_pca1bestf1 | -.0256176 .0116279 -2.20 0.028 -.0484338 -.0028014

urban | .0047342 .0068944 0.69 0.492 -.0087939 .0182623

cluster_facrad20kmlog | -.0015931 .0026169 -0.61 0.543 -.006728 .0035419

|

hh_windex5 |

Second | -.0016095 .0057102 -0.28 0.778 -.0128141 .0095951

Middle | -.0009591 .0056759 -0.17 0.866 -.0120963 .010178

Fourth | -.0091169 .0049745 -1.83 0.067 -.0188778 .0006439

Richest | .0042989 .0069914 0.61 0.539 -.0094196 .0180174

|

w_edsec | -.0076565 .0048835 -1.57 0.117 -.017239 .0019259

w_lt18birth | .0025423 .0066069 0.38 0.700 -.0104217 .0155064

inf_male | .0085114 .0031767 2.68 0.007 .002278 .0147447

inf_multiple | .0893505 .0218453 4.09 0.000 .0464857 .1322153

inf_primip | .0164391 .0054355 3.02 0.003 .0057736 .0271046

inf_lbw | .0139128 .0063002 2.21 0.027 .0015506 .026275

_cons | .0207766 .0099031 2.10 0.036 .0013448 .0402085

---------------------------------------------------------------------------------------

Instrumented: q_pca1bestf1

Instruments: urban cluster_facrad20kmlog 2.hh_windex5 3.hh_windex5 4.hh_windex5

5.hh_windex5 w_edsec w_lt18birth inf_male inf_multiple inf_primip

inf_lbw iv_difdistpca

---------------------------------------------------------------------------------------

svy: ivprobit death28 urban cluster_facrad20kmlog i.hh_windex5 w_edsec w_lt18 inf_male inf_mult inf_primi inf_lbw (q_pca1bestf1 = iv_difdistpca)

(running ivprobit on estimation sample)

Survey: Probit model with endogenous regressors

Number of strata = 55 Number of obs = 6,668

Number of PSUs = 1,120 Population size = 6,668

Design df = 1,065

F( 13, 1053) = 9.38

Prob > F = 0.0000

---------------------------------------------------------------------------------------

| Linearized

| Coef. Std. Err. t P>|t| [95% Conf. Interval]

----------------------+----------------------------------------------------------------

death28days |

q_pca1bestf1 | -.4404562 .1720858 -2.56 0.011 -.7781219 -.1027904

urban | .069555 .1584067 0.44 0.661 -.2412696 .3803796

cluster_facrad20kmlog | -.0457701 .0571281 -0.80 0.423 -.1578665 .0663262

|

hh_windex5 |

Second | -.011661 .116114 -0.10 0.920 -.2394993 .2161772

Middle | -.0381148 .1179204 -0.32 0.747 -.2694975 .1932678

Fourth | -.2965363 .1409239 -2.10 0.036 -.5730564 -.0200162

Richest | .095955 .1476068 0.65 0.516 -.1936783 .3855882

|

w_edsec | -.2020775 .1276513 -1.58 0.114 -.4525541 .0483991

w_lt18birth | .043733 .110862 0.39 0.693 -.1737997 .2612658

inf_male | .1876183 .0752888 2.49 0.013 .039887 .3353495

inf_multiple | .9600567 .128402 7.48 0.000 .708107 1.212006

inf_primip | .3647235 .0994172 3.67 0.000 .1696476 .5597993

inf_lbw | .2484395 .0933434 2.66 0.008 .0652817 .4315974

_cons | -2.032516 .1962378 -10.36 0.000 -2.417573 -1.64746

----------------------+----------------------------------------------------------------

q_pca1bestf1 |

urban | .1837955 .0291092 6.31 0.000 .1266775 .2409134

cluster_facrad20kmlog | -.0393996 .0153663 -2.56 0.010 -.0695513 -.009248

|

hh_windex5 |

Second | -.0326573 .017005 -1.92 0.055 -.0660244 .0007098

Middle | .0011807 .0173219 0.07 0.946 -.0328081 .0351696

Fourth | .0086979 .0202521 0.43 0.668 -.0310407 .0484366

Richest | .041229 .0261986 1.57 0.116 -.0101778 .0926357

|

w_edsec | .0112283 .0165149 0.68 0.497 -.0211773 .0436338

w_lt18birth | .0060357 .0208914 0.29 0.773 -.0349573 .0470286

inf_male | -.0072513 .0111831 -0.65 0.517 -.0291947 .014692

inf_multiple | .1258279 .0388049 3.24 0.001 .0496852 .2019707

inf_primip | .0564608 .0167686 3.37 0.001 .0235575 .0893641

inf_lbw | .0118878 .0158113 0.75 0.452 -.0191371 .0429127

iv_difdistpca | -.0216738 .0012332 -17.58 0.000 -.0240936 -.019254

_cons | .6581425 .0460149 14.30 0.000 .5678523 .7484327

----------------------+----------------------------------------------------------------

/athrho2_1 | .3188937 .092214 3.46 0.001 .1379519 .4998354

/lnsigma2 | -.80742 .0069925 -115.47 0.000 -.8211408 -.7936993

----------------------+----------------------------------------------------------------

corr(e.q_pca1bestf1,|

e.death28days)| .3085062 .0834374 .1370834 .4619877

sd(e.q_pca1bestf1)| .4460073 .0031187 .4399295 .452169

---------------------------------------------------------------------------------------

Instrumented: q_pca1bestf1

Instruments: urban cluster_facrad20kmlog 2.hh_windex5 3.hh_windex5 4.hh_windex5

5.hh_windex5 w_edsec w_lt18birth inf_male inf_multiple inf_primip

inf_lbw iv_difdistpca

---------------------------------------------------------------------------------------

*Robustness check 5: Continuous quality index

*Exposure: standardized facility quality index

*IV: Difference between distance to nearest delivery facility and nearest high quality facility

svy: ivregress 2sls death28 urban cluster_facrad20kmlog i.hh_windex5 w_edsec w_lt18 inf_male inf_mult inf_primi inf_lbw (q_indexstdf1 = iv_difdistbest)

(running ivregress on estimation sample)

Survey: Instrumental variables (2SLS) regression

Number of strata = 55 Number of obs = 6,668

Number of PSUs = 1,120 Population size = 6,668

Design df = 1,065

F( 13, 1053) = 3.82

Prob > F = 0.0000

R-squared = 0.0034

---------------------------------------------------------------------------------------

| Linearized

death28days | Coef. Std. Err. t P>|t| [95% Conf. Interval]

----------------------+----------------------------------------------------------------

q_indexstdf1 | -.0270115 .0137678 -1.96 0.050 -.0540266 3.53e-06

urban | .0099207 .0091791 1.08 0.280 -.0080906 .0279319

cluster_facrad20kmlog | -.0015782 .0026592 -0.59 0.553 -.006796 .0036397

|

hh_windex5 |

Second | -.001453 .0057499 -0.25 0.801 -.0127354 .0098295

Middle | -.0007073 .00568 -0.12 0.901 -.0118526 .010438

Fourth | -.0079617 .0050258 -1.58 0.113 -.0178234 .0019

Richest | .004775 .0070211 0.68 0.497 -.0090018 .0185517

|

w_edsec | -.0071476 .0049118 -1.46 0.146 -.0167855 .0024904

w_lt18birth | .0026027 .0066339 0.39 0.695 -.0104143 .0156198

inf_male | .008828 .0031961 2.76 0.006 .0025567 .0150993

inf_multiple | .091877 .0221822 4.14 0.000 .0483511 .1354028

inf_primip | .0174066 .00559 3.11 0.002 .0064379 .0283752

inf_lbw | .0145582 .0063633 2.29 0.022 .0020721 .0270443

_cons | .0342748 .0150738 2.27 0.023 .0046971 .0638526

---------------------------------------------------------------------------------------

Instrumented: q_indexstdf1

Instruments: urban cluster_facrad20kmlog 2.hh_windex5 3.hh_windex5 4.hh_windex5

5.hh_windex5 w_edsec w_lt18birth inf_male inf_multiple inf_primip

inf_lbw iv_difdistbest

---------------------------------------------------------------------------------------

svy: ivprobit death28 urban cluster_facrad20kmlog i.hh_windex5 w_edsec w_lt18 inf_male inf_mult inf_primi inf_lbw (q_indexstdf1 = iv_difdistbest)

(running ivprobit on estimation sample)

Survey: Probit model with endogenous regressors

Number of strata = 55 Number of obs = 6,668

Number of PSUs = 1,120 Population size = 6,668

Design df = 1,065

F( 13, 1053) = 10.07

Prob > F = 0.0000

---------------------------------------------------------------------------------------

| Linearized

| Coef. Std. Err. t P>|t| [95% Conf. Interval]

----------------------+----------------------------------------------------------------

death28days |

q_indexstdf1 | -.4514791 .1937044 -2.33 0.020 -.8315647 -.0713935

urban | .1449253 .1800935 0.80 0.421 -.2084531 .4983037

cluster_facrad20kmlog | -.0455344 .0565684 -0.80 0.421 -.1565326 .0654638

|

hh_windex5 |

Second | -.0118532 .113801 -0.10 0.917 -.2351528 .2114464

Middle | -.0355167 .1146953 -0.31 0.757 -.260571 .1895377

Fourth | -.2757037 .137751 -2.00 0.046 -.5459978 -.0054095

Richest | .1020628 .1446224 0.71 0.481 -.1817144 .3858401

|

w_edsec | -.1839494 .1250179 -1.47 0.141 -.4292589 .06136

w_lt18birth | .0418597 .108038 0.39 0.698 -.1701318 .2538512

inf_male | .1890099 .0735224 2.57 0.010 .0447446 .3332752

inf_multiple | .9757953 .1258849 7.75 0.000 .7287847 1.222806

inf_primip | .3722468 .0969294 3.84 0.000 .1820526 .562441

inf_lbw | .2523011 .0913795 2.76 0.006 .0729967 .4316055

_cons | -1.746277 .2979073 -5.86 0.000 -2.330829 -1.161725

----------------------+----------------------------------------------------------------

q_indexstdf1 |

urban | .381289 .0390343 9.77 0.000 .3046961 .457882

cluster_facrad20kmlog | -.0255808 .0195052 -1.31 0.190 -.0638538 .0126922

|

hh_windex5 |

Second | -.0281683 .0252842 -1.11 0.266 -.0777808 .0214441

Middle | .004935 .0244612 0.20 0.840 -.0430626 .0529326

Fourth | .0452432 .0291159 1.55 0.121 -.0118878 .1023742

Richest | .0499506 .038142 1.31 0.191 -.0248914 .1247926

|

w_edsec | .0270176 .0232639 1.16 0.246 -.0186308 .0726659

w_lt18birth | .0046822 .0290722 0.16 0.872 -.0523631 .0617275

inf_male | .0050813 .015147 0.34 0.737 -.0246401 .0348027

inf_multiple | .2165498 .0484622 4.47 0.000 .1214575 .3116421

inf_primip | .0895649 .0232444 3.85 0.000 .0439549 .1351749

inf_lbw | .0348533 .0218562 1.59 0.111 -.0080328 .0777395

iv_difdistbest | -.0186845 .0018616 -10.04 0.000 -.0223374 -.0150317

_cons | 1.07956 .060352 17.89 0.000 .961138 1.197983

----------------------+----------------------------------------------------------------

/athrho2_1 | .3925083 .1441601 2.72 0.007 .1096382 .6753784

/lnsigma2 | -.4750744 .0153635 -30.92 0.000 -.5052205 -.4449283

----------------------+----------------------------------------------------------------

corr(e.q_indexstdf1,|

e.death28days)| .3735206 .1240472 .109201 .5885067

sd(e.q_indexstdf1)| .6218388 .0095536 .6033725 .6408703

---------------------------------------------------------------------------------------

Instrumented: q_indexstdf1

Instruments: urban cluster_facrad20kmlog 2.hh_windex5 3.hh_windex5 4.hh_windex5

5.hh_windex5 w_edsec w_lt18birth inf_male inf_multiple inf_primip

inf_lbw iv_difdistbest

---------------------------------------------------------------------------------------

*Robustness check 5: Alternative quality metric

*Exposure: Composite measure of structural, process, and observed quality

*IV: Differential distance using top 25% of facilities on composite measure

ivregress 2sls death28 urban cluster_facrad20kmlog i.hh_windex5 w_edsec w_lt18 inf_male

inf_mult inf_primi inf_lbw (q_stprobsbestf1 = iv_difdistcomp) , vce(cluster hh1)

Instrumental variables (2SLS) regression Number of obs = 4,171

Wald chi2(13) = 38.35

Prob > chi2 = 0.0003

R-squared = 0.0266

Root MSE = .13859

(Std. Err. adjusted for 933 clusters in hh1)

---------------------------------------------------------------------------------------

| Robust

death28days | Coef. Std. Err. z P>|z| [95% Conf. Interval]

----------------------+----------------------------------------------------------------

q_stprobsbestf1 | -.0164468 .0110707 -1.49 0.137 -.038145 .0052514

urban | .0025296 .0074431 0.34 0.734 -.0120586 .0171178

cluster_facrad20kmlog | -.0008937 .0036902 -0.24 0.809 -.0081264 .006339

|

hh_windex5 |

Second | .0060458 .0076178 0.79 0.427 -.0088847 .0209764

Middle | .0027824 .0070232 0.40 0.692 -.0109829 .0165476

Fourth | -.0055321 .0066948 -0.83 0.409 -.0186537 .0075896

Richest | .0074594 .0090953 0.82 0.412 -.0103672 .0252859

|

w_edsec | -.0090752 .0068888 -1.32 0.188 -.0225769 .0044265

w_lt18birth | -.0002074 .0091398 -0.02 0.982 -.0181212 .0177063

inf_male | .0095485 .0043622 2.19 0.029 .0009987 .0180982

inf_multiple | .0970835 .0265954 3.65 0.000 .0449574 .1492095

inf_primip | .0191717 .0070678 2.71 0.007 .0053191 .0330243

inf_lbw | .0181343 .0084346 2.15 0.032 .0016028 .0346658

_cons | .0112229 .0121732 0.92 0.357 -.0126361 .0350819

---------------------------------------------------------------------------------------

Instrumented: q_stprobsbestf1

Instruments: urban cluster_facrad20kmlog 2.hh_windex5 3.hh_windex5 4.hh_windex5

5.hh_windex5 w_edsec w_lt18birth inf_male inf_multiple inf_primip

inf_lbw iv_difdistcomp

ivprobit death28 urban cluster_facrad20kmlog i.hh_windex5 w_edsec w_lt18 inf_male inf_mult inf_primi inf_lbw (q_stprobsbestf1 = iv_difdistcomp) , vce(cluster hh1)

Fitting exogenous probit model

Iteration 0: log likelihood = -411.17634

Iteration 1: log likelihood = -370.14703

Iteration 2: log likelihood = -364.03769

Iteration 3: log likelihood = -364.03112

Iteration 4: log likelihood = -364.03112

Fitting full model

Iteration 0: log pseudolikelihood = -2690.6036

Iteration 1: log pseudolikelihood = -2690.5918

Iteration 2: log pseudolikelihood = -2690.5918

Probit model with endogenous regressors Number of obs = 4,171

Wald chi2(13) = 86.25

Log pseudolikelihood = -2690.5918 Prob > chi2 = 0.0000

(Std. Err. adjusted for 933 clusters in hh1)

-----------------------------------------------------------------------------------------

| Robust

| Coef. Std. Err. z P>|z| [95% Conf. Interval]

------------------------+----------------------------------------------------------------

q_stprobsbestf1 | -.3040278 .1871173 -1.62 0.104 -.6707709 .0627153

urban | .0324212 .1656811 0.20 0.845 -.2923078 .3571503

cluster_facrad20kmlog | -.0367333 .0683721 -0.54 0.591 -.1707402 .0972735

|

hh_windex5 |

Second | .1573171 .1503028 1.05 0.295 -.137271 .4519052

Middle | .0572298 .1509141 0.38 0.705 -.2385565 .3530161

Fourth | -.2035912 .1822879 -1.12 0.264 -.5608689 .1536865

Richest | .1759456 .1838822 0.96 0.339 -.1844569 .5363481

|

w_edsec | -.2152855 .1588734 -1.36 0.175 -.5266716 .0961006

w_lt18birth | .0138631 .1394458 0.10 0.921 -.2594456 .2871719

inf_male | .1913479 .0944419 2.03 0.043 .0062452 .3764506

inf_multiple | 1.010916 .1495774 6.76 0.000 .7177495 1.304082

inf_primip | .4002847 .118627 3.37 0.001 .16778 .6327893

inf_lbw | .2887218 .1122543 2.57 0.010 .0687074 .5087362

_cons | -2.237658 .2315895 -9.66 0.000 -2.691566 -1.783751

------------------------+----------------------------------------------------------------

corr(e.q_stprobsbestf1,|

e.death28days)| .2281554 .0916493 .0427232 .3983921

sd(e.q_stprobsbestf1)| .4226782 .0052644 .4124852 .4331232

-----------------------------------------------------------------------------------------

Instrumented: q_stprobsbestf1

Instruments: urban cluster_facrad20kmlog 2.hh_windex5 3.hh_windex5 4.hh_windex5

5.hh_windex5 w_edsec w_lt18birth inf_male inf_multiple inf_primip

inf_lbw iv_difdistcomp

-----------------------------------------------------------------------------------------

Wald test of exogeneity (corr = 0): chi2(1) = 5.77 Prob > chi2 = 0.0163

*Alternative models

*1: Hospital

*Exposure: hospital delivery

*IV: Differential distance to hospital

svy: ivregress 2sls death28 urban cluster_facrad20kmlog i.hh_windex5 w_edsec w_lt18 inf_male inf_mult inf_primi inf_lbw (del_hosp = iv_difdisthosp)

(running ivregress on estimation sample)

Survey: Instrumental variables (2SLS) regression

Number of strata = 55 Number of obs = 6,697

Number of PSUs = 1,120 Population size = 6,697

Design df = 1,065

F( 13, 1053) = 3.88

Prob > F = 0.0000

R-squared = 0.0172

---------------------------------------------------------------------------------------

| Linearized

death28days | Coef. Std. Err. t P>|t| [95% Conf. Interval]

----------------------+----------------------------------------------------------------

del_hospital | -.0203083 .0165046 -1.23 0.219 -.0526936 .012077

urban | .0036384 .0078891 0.46 0.645 -.0118416 .0191185

cluster_facrad20kmlog | -.0028519 .0026939 -1.06 0.290 -.0081378 .002434

|

hh_windex5 |

Second | -.000744 .0056844 -0.13 0.896 -.0118979 .0104099

Middle | -.0001081 .0055497 -0.02 0.984 -.0109977 .0107815

Fourth | -.0077571 .0048997 -1.58 0.114 -.0173712 .001857

Richest | .0060255 .0069915 0.86 0.389 -.0076932 .0197441

|

w_edsec | -.0070761 .0048509 -1.46 0.145 -.0165946 .0024424

w_lt18birth | .0027692 .0065383 0.42 0.672 -.0100602 .0155985

inf_male | .0087776 .003162 2.78 0.006 .0025732 .014982

inf_multiple | .0906732 .0219755 4.13 0.000 .0475529 .1337934

inf_primip | .0167635 .0056469 2.97 0.003 .0056831 .0278438

inf_lbw | .0136678 .0062508 2.19 0.029 .0014025 .0259331

_cons | .0194299 .0103897 1.87 0.062 -.0009566 .0398165

---------------------------------------------------------------------------------------

Instrumented: del_hospital

Instruments: urban cluster_facrad20kmlog 2.hh_windex5 3.hh_windex5 4.hh_windex5

5.hh_windex5 w_edsec w_lt18birth inf_male inf_multiple inf_primip

inf_lbw iv_difdisthosp

---------------------------------------------------------------------------------------

. svy: ivprobit death28 urban cluster_facrad20kmlog i.hh_windex5 w_edsec w_lt18 inf_male inf_mult inf_primi inf_lbw (del_hosp = iv_difdisthosp)

(running ivprobit on estimation sample)

Survey: Probit model with endogenous regressors

Number of strata = 55 Number of obs = 6,697

Number of PSUs = 1,120 Population size = 6,697

Design df = 1,065

F( 13, 1053) = 8.93

Prob > F = 0.0000

---------------------------------------------------------------------------------------

| Linearized

| Coef. Std. Err. t P>|t| [95% Conf. Interval]

----------------------+----------------------------------------------------------------

death28days |

del_hospital | -.3371302 .2904671 -1.16 0.246 -.907083 .2328225

urban | .0431698 .1761022 0.25 0.806 -.3023769 .3887164

cluster_facrad20kmlog | -.0812524 .0596071 -1.36 0.173 -.198213 .0357081

|

hh_windex5 |

Second | .0043467 .1178658 0.04 0.971 -.226929 .2356223

Middle | -.0223088 .1177073 -0.19 0.850 -.2532734 .2086558

Fourth | -.2679414 .1412339 -1.90 0.058 -.5450698 .009187

Richest | .1222601 .1477965 0.83 0.408 -.1677453 .4122655

|

w_edsec | -.1918539 .1304677 -1.47 0.142 -.4478567 .064149

w_lt18birth | .0450235 .1107153 0.41 0.684 -.1722213 .2622683

inf_male | .1933429 .0757546 2.55 0.011 .0446978 .3419881

inf_multiple | .9972041 .1318178 7.57 0.000 .738552 1.255856

inf_primip | .3790804 .1008242 3.76 0.000 .1812436 .5769171

inf_lbw | .2461635 .0939281 2.62 0.009 .0618583 .4304687

_cons | -2.056084 .2343843 -8.77 0.000 -2.515991 -1.596176

----------------------+----------------------------------------------------------------

del_hospital |

urban | .219828 .0286827 7.66 0.000 .1635471 .2761089

cluster_facrad20kmlog | -.0440857 .0131534 -3.35 0.001 -.0698953 -.0182762

|

hh_windex5 |

Second | -.0121237 .0164523 -0.74 0.461 -.0444063 .0201589

Middle | .0105369 .0168051 0.63 0.531 -.022438 .0435118

Fourth | .022809 .0201927 1.13 0.259 -.0168131 .062431

Richest | .0774207 .0259079 2.99 0.003 .0265843 .1282571

|

w_edsec | .0313548 .0166839 1.88 0.060 -.0013823 .064092

w_lt18birth | .0073047 .0208867 0.35 0.727 -.0336791 .0482885

inf_male | .0086953 .0112679 0.77 0.440 -.0134146 .0308051

inf_multiple | .1958709 .0392679 4.99 0.000 .1188196 .2729222

inf_primip | .0796037 .0172684 4.61 0.000 .0457198 .1134876

inf_lbw | .0073849 .0157301 0.47 0.639 -.0234807 .0382504

iv_difdisthosp | -.0151612 .0010437 -14.53 0.000 -.0172092 -.0131132

_cons | .5575481 .0423952 13.15 0.000 .4743604 .6407358

----------------------+----------------------------------------------------------------

/athrho2_1 | .2616639 .1434835 1.82 0.068 -.0198786 .5432063

/lnsigma2 | -.8010444 .0073909 -108.38 0.000 -.8155468 -.786542

----------------------+----------------------------------------------------------------

corr(e.del_hospital,|

e.death28days)| .2558511 .1340911 -.0198759 .4954112

sd(e.del_hospital)| .4488599 .0033175 .4423973 .4554169

---------------------------------------------------------------------------------------

Instrumented: del_hospital

Instruments: urban cluster_facrad20kmlog 2.hh_windex5 3.hh_windex5 4.hh_windex5

5.hh_windex5 w_edsec w_lt18birth inf_male inf_multiple inf_primip

inf_lbw iv_difdisthosp

---------------------------------------------------------------------------------------

*2: Okeke index of facility capacity

*Exposure: 7-item index of facility capacity

*IV: Differential distance using top 25% of facilities on capacity index

svy: ivregress 2sls death28 urban cluster_facrad20kmlog i.hh_windex5 w_edsec w_lt18 inf_male inf_mult inf_primi inf_lbw (q_capacitybestf1 = iv_difdistqc)

(running ivregress on estimation sample)

Survey: Instrumental variables (2SLS) regression

Number of strata = 55 Number of obs = 6,668

Number of PSUs = 1,120 Population size = 6,668

Design df = 1,065

F( 13, 1053) = 3.92

Prob > F = 0.0000

R-squared = 0.0195

---------------------------------------------------------------------------------------

| Linearized

death28days | Coef. Std. Err. t P>|t| [95% Conf. Interval]

----------------------+----------------------------------------------------------------

q_capacitybestf1 | -.0189712 .0140845 -1.35 0.178 -.0466077 .0086654

urban | .0038724 .007732 0.50 0.617 -.0112993 .019044

cluster_facrad20kmlog | -.0018528 .0026432 -0.70 0.483 -.0070393 .0033338

|

hh_windex5 |

Second | -.0007891 .0056626 -0.14 0.889 -.0119003 .010322

Middle | -.0000994 .0056388 -0.02 0.986 -.0111639 .010965

Fourth | -.0081727 .0050351 -1.62 0.105 -.0180525 .0017071

Richest | .0052868 .0070167 0.75 0.451 -.0084813 .019055

|

w_edsec | -.007689 .0048594 -1.58 0.114 -.017224 .001846

w_lt18birth | .0032003 .0065003 0.49 0.623 -.0095546 .0159553

inf_male | .0086228 .0031609 2.73 0.006 .0024205 .0148251

inf_multiple | .088907 .0218737 4.06 0.000 .0459867 .1318274

inf_primip | .015572 .005343 2.91 0.004 .005088 .026056

inf_lbw | .0140247 .0062744 2.24 0.026 .0017131 .0263363

_cons | .018863 .0100395 1.88 0.061 -.0008364 .0385625

---------------------------------------------------------------------------------------

Instrumented: q_capacitybestf1

Instruments: urban cluster_facrad20kmlog 2.hh_windex5 3.hh_windex5 4.hh_windex5

5.hh_windex5 w_edsec w_lt18birth inf_male inf_multiple inf_primip

inf_lbw iv_difdistqc

---------------------------------------------------------------------------------------

svy: ivprobit death28 urban cluster_facrad20kmlog i.hh_windex5 w_edsec w_lt18 inf_male inf_mult inf_primi inf_lbw (q_capacitybestf1 = iv_difdistqc)

(running ivprobit on estimation sample)

Survey: Probit model with endogenous regressors

Number of strata = 55 Number of obs = 6,668

Number of PSUs = 1,120 Population size = 6,668

Design df = 1,065

F( 13, 1053) = 8.84

Prob > F = 0.0000

------------------------------------------------------------------------------------------

| Linearized

| Coef. Std. Err. t P>|t| [95% Conf. Interval]

-------------------------+----------------------------------------------------------------

death28days |

q_capacitybestf1 | -.3247696 .2184671 -1.49 0.137 -.7534443 .1039052

urban | .0470927 .1691455 0.28 0.781 -.2848036 .378989

cluster_facrad20kmlog | -.0577611 .0608242 -0.95 0.343 -.1771101 .0615878

|

hh_windex5 |

Second | -.0002116 .1179166 -0.00 0.999 -.2315868 .2311636

Middle | -.0257468 .1190811 -0.22 0.829 -.2594071 .2079135

Fourth | -.2898003 .1450453 -2.00 0.046 -.5744074 -.0051933

Richest | .1151486 .1489651 0.77 0.440 -.1771498 .407447

|

w_edsec | -.1980218 .1295914 -1.53 0.127 -.4523054 .0562617

w_lt18birth | .0610013 .1101174 0.55 0.580 -.1550704 .277073

inf_male | .1936156 .0761534 2.54 0.011 .0441879 .3430434

inf_multiple | .9753948 .1296733 7.52 0.000 .7209505 1.229839

inf_primip | .3629419 .0994434 3.65 0.000 .1678148 .5580691

inf_lbw | .2514721 .0945155 2.66 0.008 .0660143 .4369299

_cons | -2.090673 .2069397 -10.10 0.000 -2.496729 -1.684617

-------------------------+----------------------------------------------------------------

q_capacitybestf1 |

urban | .2492725 .0269023 9.27 0.000 .196485 .30206

cluster_facrad20kmlog | -.0148048 .0145548 -1.02 0.309 -.043364 .0137545

|

hh_windex5 |

Second | -.0123654 .0173396 -0.71 0.476 -.046389 .0216583

Middle | .0206662 .0178178 1.16 0.246 -.0142958 .0556283

Fourth | .0159295 .0200168 0.80 0.426 -.0233473 .0552063

Richest | .0581753 .0261677 2.22 0.026 .0068291 .1095215

|

w_edsec | .0087152 .0157043 0.55 0.579 -.0220997 .0395301

w_lt18birth | .0206744 .0210019 0.98 0.325 -.0205353 .0618842

inf_male | -.0044805 .0110642 -0.40 0.686 -.0261907 .0172297

inf_multiple | .1235553 .0361754 3.42 0.001 .0525722 .1945383

inf_primip | .0413618 .0168488 2.45 0.014 .0083011 .0744225

inf_lbw | .0179201 .0153387 1.17 0.243 -.0121774 .0480175

iv_difdistqc | -.0250606 .0015775 -15.89 0.000 -.028156 -.0219652

_cons | .601115 .0444785 13.51 0.000 .5138394 .6883905

-------------------------+----------------------------------------------------------------

/athrho2_1 | .2388868 .1092379 2.19 0.029 .0245409 .4532327

/lnsigma2 | -.8139322 .0079342 -102.59 0.000 -.8295007 -.7983637

-------------------------+----------------------------------------------------------------

corr(e.q_capacitybestf1,|

e.death28days)| .234444 .1032337 .024536 .4245527

sd(e.q_capacitybestf1)| .4431122 .0035157 .4362671 .4500648

------------------------------------------------------------------------------------------

Instrumented: q_capacitybestf1

Instruments: urban cluster_facrad20kmlog 2.hh_windex5 3.hh_windex5 4.hh_windex5

5.hh_windex5 w_edsec w_lt18birth inf_male inf_multiple inf_primip inf_lbw

iv_difdistqc

------------------------------------------------------------------------------------------

**Supplemental References**

1. Pizer SD. Falsification Testing of Instrumental Variables Methods for Comparative Effectiveness Research. Health Serv Res. 2016;51(2):790-811.

2. Swanson SA, Hernan MA. Commentary: how to report instrumental variable analyses (suggestions welcome). Epidemiology. 2013;24(3):370-4.
